# The acute effect of heat exposure on forearm macro‐ and microvascular function: Impact of measurement timing, heating modality and biological sex

**DOI:** 10.1113/EP090732

**Published:** 2022-12-19

**Authors:** Georgia K. Chaseling, Amélie Debray, Hugo Gravel, Nicholas Ravanelli, Audrey‐Ann Bartlett, Daniel Gagnon

**Affiliations:** ^1^ Montreal Heart Institute Montréal Québec Canada; ^2^ Department of Pharmacology and Physiology Faculty of Medicine Université de Montréal Montréal Québec Canada; ^3^ Department of Medicine Faculty of Medicine Université de Montréal Montréal Québec Canada; ^4^ School of Kinesiology and Exercise Science Faculty of Medicine Université de Montréal Montréal Québec Canada; ^5^ School of Kinesiology Lakehead University Thunder Bay Ontario Canada

**Keywords:** cardiovascular, endothelial, heat, shear, temperature, vascular

## Abstract

The aim of this study was to gain a better understanding of the acute effect of heat exposure on brachial artery flow‐mediated dilatation (FMD) and postocclusion reactive hyperaemia (PORH) by: characterizing the time course of changes post‐heating; comparing forearm and whole‐body heating; determining the impact of forearm heating during whole‐body heating; and comparing males and females. Twenty adults (11 males and nine females; 28 ± 6 years of age) underwent two forearm [10 min electric blanket (EB) or 30 min hot water immersion (WI)] and two whole‐body [60 min water‐perfused suit with forearm covered (WBH‐C) or uncovered (WBH‐U)] heating modalities. The FMD and PORH were measured before and after (≤5, 30, 60, 90 and 120 min) heating. The FMD increased from baseline 30 min after EB, and 30 and 90 min after WI. In contrast, FMD decreased from baseline immediately after both WBH modalities. Peak PORH increased immediately after WI and both WBH modalities. Total PORH did not differ after WI, whereas it decreased immediately after both WBH modalities. Covering the forearm during WBH did not alter acute changes in FMD or PORH. Changes in FMD and PORH did not differ statistically between males and females during each heating modality, although the observed differences could not always be considered equivalent. These results demonstrate that the acute effect of heat exposure on brachial artery FMD and PORH is: (1) transient and short lasting; (2) different between forearm heating and WBH; (3) unaffected by direct forearm heating during WBH; and (4) not different but not always equivalent between males and females.

## Abstract (highlights)

- **What is the central question of this study?**
  Do measurement timing, heating modality and biological sex modulate the acute effect of heat exposure on brachial artery flow‐mediated dilatation and postocclusion reactive hyperaemia?
- **What is the main finding and its importance?**
  The acute effect of heat exposure on brachial artery flow‐mediated dilatation and postocclusion reactive hyperaemia is: (1) transient and short lasting; (2) different between forearm and whole‐body heating; (3) unaffected by forearm heating during whole‐body heating; and (4) not different but not always equivalent between males and females. These findings provide a useful basis for future studies to investigate the acute effect of heat exposure on vascular function.

## 1 INTRODUCTION

In recent years, there has been a renewed interest in the potential cardiovascular health benefits of repeated heat exposure (i.e., heat therapy). This interest is attributable, in part, to the effect of heat exposure on macro‐ and microvascular function, measured by flow‐mediated dilatation (FMD) and vascular conductance during postocclusion reactive hyperaemia (PORH), respectively. In some interventional studies, improvements in FMD (Bailey et al., 2016; Brunt, Howard et al., 2016; Imamura et al., 2001; Kihara et al., 2002; Naylor et al., 2011) or PORH (Brunt, Howard et al., 2016) have been observed after repeated bouts of heat exposure, although these are not universal findings (Akerman et al., 2019; McGarity‐Shipley et al., 2021). To gain a better understanding of how heat exposure might modulate vascular function, studies have also considered the acute effect of heat exposure on FMD and/or PORH. It has been hypothesized that the cardiovascular adjustments following acute heat exposure might predict longer‐term adaptations (Romero et al., 2022). However, the acute effect of heat exposure on FMD and PORH has proved variable, with some studies observing an improvement in FMD (Cheng et al., 2019, 2021; Coombs et al., 2021; Gravel et al., 2020; Romero et al., 2017; Tinken et al., 2009) or PORH (Cheng et al., 2021; Romero et al., 2017), whereas others did not observe an acute change in FMD (Alali et al., 2020; Behzadi et al., 2022; Brunt, Jeckell et al., 2016; Coombs et al., 2019; Engelland et al., 2020; Gravel et al., 2019; Thomas et al., 2016) or PORH (Behzadi et al., 2022; Brunt, Jeckell et al., 2016; Engelland et al., 2020; Gravel et al., 2020, 2019).

The factors that underlie the variable effect of heat exposure on macro‐ and microvascular function are unknown. One possibility relates to considerable heterogeneity in the study designs used to date. First, previous studies measured vascular function at a single time point following heat exposure, and these time points have ranged from during heating to immediately after heating to ≤60 min post‐heating. Studies in which an acute effect of heating was observed typically involved measurements within 30–45 min post‐heating (Cheng et al., 2019, 2021; Coombs et al., 2021; Gravel et al., 2020; Romero et al., 2017; Tinken et al., 2009), whereas no effect was observed when measurements were performed ∼45–60 min post‐heating (Behzadi et al., 2022; Brunt, Jeckell et al., 2016; Engelland et al., 2020; Gravel et al., 2019). Given it is unknown whether any acute change in vascular function after heating is transient or long lasting, it is possible that previous studies involving measurements within a longer time frame missed the window to detect an acute change in macro‐ and/or microvascular function.

Second, previous studies used limb or whole‐body heating modalities. In studies that used limb heating, an acute improvement in FMD (Cheng et al., 2019, 2021; Romero et al., 2017; Tinken et al., 2009) or PORH (Cheng et al., 2021; Romero et al., 2017) was generally observed, whereas in studies that used a whole‐body heating modality, an acute effect of heat exposure was generally not observed (Alali et al., 2020; Behzadi et al., 2022; Brunt, Jeckell et al., 2016; Gravel et al., 2019; Thomas et al., 2016). This discrepancy is noteworthy, considering that whole‐body heating protocols are typically associated with greater elevations in shear stress and core temperature, which are considered important mediators of the longer‐term effect of heat exposure on vascular function (Brunt & Minson, 2021; Cheng & MacDonald, 2019). One consideration when comparing limb versus whole‐body heating modalities is that the limb from which vascular function is measured is not typically exposed to the heating stimulus during whole‐body heating (Alali et al., 2020; Brunt, Jeckell et al., 2016; Coombs et al., 2021; Thomas et al., 2016). An increase in local skin temperature is required to observe cutaneous microvascular adaptations in response to repeated lower‐limb heating (Carter, Spence, Atkinson, Pugh, Cable et al., 2014). Therefore, it is possible that direct heating of a limb is also needed to observe an acute improvement in FMD and/or PORH.

Third, it remains unknown whether biological sex contributes to the variable effect of acute heat exposure on vascular function, because previous studies have reported results from males only (Alali et al., 2020; Cheng et al., 2019; Coombs et al., 2019; Tinken et al., 2009), combined groups of males and females (Behzadi et al., 2022; Brunt, Jeckell et al., 2016; Cheng et al., 2021; Engelland et al., 2020; Gravel et al., 2020, 2019; Romero et al., 2017; Thomas et al., 2016) or studied females only (McGarity‐Shipley et al., 2021). It is possible that biological sex modulates the acute effect of heat exposure on vascular function, because females display greater increases in shear stress and/or core temperature for a given heating stimulus (Larson et al., 2021).

The general objective of this study was to gain a better understanding of the acute effect of heat exposure on brachial artery FMD and PORH. Specifically, we aimed to address the following four knowledge gaps:

1. What is the time course of any acute change in brachial artery FMD and PORH following limb and whole‐body heating? We tested the hypothesis that heat exposure would lead to a rapid and transient improvement in brachial artery FMD and PORH.
2. Does the acute effect of heat exposure on brachial artery FMD and PORH differ between limb and whole‐body heating? We tested the hypothesis that the magnitude of change in brachial artery FMD and PORH would be greater after limb heating.
3. Is exposure of the limb from which FMD and PORH are measured required to observe an acute improvement in these variables following whole‐body heating? We tested the hypothesis that a greater change in brachial artery FMD and PORH would be observed when the forearm is directly exposed to the heating stimulus during whole‐body heating.
4. Does biological sex modulate the acute effect of heat exposure on brachial artery FMD and PORH? We explored the hypothesis that females display a greater change in brachial artery FMD and PORH following heat exposure.

## 2 METHODS

### 2.1 Ethical approval

This study was approved by the Research Ethics and New Technologies Development Committee of the Montreal Heart Institute (approval no. 2020‐2657). All participants provided their written informed consent. The study conformed to the standard set by the *Declaration of Helsinki*, except for registration in a database.

### 2.2 Participants

Twenty‐one adults were approached to participate in this study; one was lost to follow‐up, resulting in a final sample size of 20 participants (11 males: 30 ± 6 years of age, 70 ± 9 kg, 1.7 ± 0.1 m, 22.3 ± 2.3 kg/m^2^; and nine females: 25 ± 4 years of age, 57 ± 7 kg, 1.6 ± 0.0 m, 20.1 ± 3.1 kg/m^2^). Inclusion criteria were as follows: age between 18 and 45 years; body mass index <30 kg/m^2^; resting blood pressure <140/<90 mmHg; and non‐smoker. Exclusion criteria included the following: a history of any chronic disease and/or a prescription of medications for the treatment of such diseases. For female participants, eight participated between days 1 and 10 after their self‐reported onset of menses, and two had an intrauterine device, reporting no regular cycle. Eligibility was determined during a screening visit, during which participants filled out a detailed medical history and lifestyle questionnaire, followed by anthropometric measurements and a resting ECG and blood pressure measurement.

### 2.3 Study design

The study involved measurements of brachial artery FMD and PORH before and after one of four heating modalities: (1) forearm heating with an electric blanket; (2) forearm hot water immersion; (3) whole‐body heating with the arm on which FMD and PORH were measured exposed to the heating stimulus; and (4) whole‐body heating with the arm on which FMD and PORH were measured not exposed to the heating stimulus. The limb heating modalities (electric blanket and forearm hot water immersion) were chosen because, at the time of designing this study (31 July 2019), these were the only heating interventions for which an acute improvement in brachial artery FMD had been reported (Cheng et al., 2019; Tinken et al., 2009). The whole‐body heating modalities were performed with a water‐perfused suit rather than hot water immersion, because this approach avoided any potential hydrostatic effects and allowed for rapid measurements of brachial artery FMD and PORH following heat exposure without postural changes. The participants were exposed to each heating modality on separate days in a randomized, counterbalanced order. The study visits were performed at the same time of day for a given participant and were separated by a minimum of 48 h. Participants were asked to avoid strenuous exercise (for 24 h), caffeine and alcohol (for 12 h) and to fast (for 6 h) before each visit (Thijssen et al., 2019).

### 2.4 Study visits

On arrival in the laboratory, participants provided a urine sample to measure urine specific gravity (PAL‐10S; Atago). If urine specific gravity was >1.025, participants were provided with 500 ml of water before proceeding with the protocol. Participants were then instrumented and baseline data recorded for 10 min before a first brachial artery FMD and PORH measurement. Subsequently, participants underwent one of the four heating modalities.

Forearm heating with an electric blanket consisted of wrapping the right forearm from the wrist to the elbow in an electric blanket (Sunbeam XpressHeat heating pad, Brampton, ON, Canada) for 10 min to replicate a previous study that reported an acute improvement in FMD with this approach (Cheng et al., 2019). Forearm water immersion consisted of submerging the right forearm in 40°C water to just above the elbow for 30 min to replicate a previous study that reported an acute improvement in FMD with this approach (Tinken et al., 2009). The temperature of the water was measured with a thermocouple and held constant by adding hot water as needed. The whole‐body heating modalities were performed with a tube‐lined suit (COOLTUBEsuit; Med‐Eng, Ottawa, ON, Canada) that covered the entire body, except for the head, hands and feet. For one visit, both arms were covered by the suit such that the arm on which FMD and PORH measurements were performed was directly exposed to the heating stimulus. For the other visit, the right sleeve was rolled up such that the arm on which FMD and PORH measurements were performed was not exposed to the heating stimulus. During baseline measurements, water at 34°C circulated (AD07H200; PolyScience, Niles, IL, USA) through the suit, whereas water at 50°C circulated through the suit for 60 min to elicit heat exposure. A duration of 60 min was chosen to replicate the duration commonly used during hot water immersion (Brunt, Jeckell et al., 2016). At the end of heating, the temperature of the water circulating through the suit was reduced to 34°C for the duration of the post‐heating period.

Following each heating modality, brachial artery FMD and PORH were measured within 5 min of the end of heating and at 30, 60, 90 and 120 min post‐heating. Participants remained in the supine position for all heating modalities, except for the visit that involved forearm water immersion, during which participants sat in an upright position to replicate the previous study that observed an acute improvement in brachial artery FMD with this approach (Tinken et al., 2009).

### 2.5 Measurements

Heart rate was obtained from lead II of a five‐lead ECG (Solar i8000; GE Healthcare). Systolic and diastolic blood pressures were measured using ECG‐gated auscultation of the brachial artery (Tango M2; SunTech Medical). Rectal temperature was measured using a general‐purpose paediatric thermistor (TM400; Covidien, Mansfield, MA, USA) self‐inserted to a depth of ∼15 cm past the anal sphincter (Lee et al., 2010). Skin temperature was measured at four sites across the left side of the body using T‐type thermocouples secured to the skin using surgical tape. A wireless temperature sensor (iButtons; Embedded Data Systems) was also taped on the forearm of the arm on which FMD measurements were performed. All FMD and PORH measurements for a given participant were performed by a single trained researcher according to expert guidelines (Thijssen et al., 2019).

Brachial artery diameter and blood velocity were measured simultaneously with a high‐resolution Doppler ultrasound machine (uSmart3300; Terason) equipped with a 4–15 MHz linear array transducer at an insonation angle of 60°. The transducer remained in a fixed position using a mechanical arm. One‐minute recordings of brachial artery diameter and blood velocity were performed at 5 and 10 min during electric blanket heating, at 15 and 30 min during forearm hot water immersion and at 15, 30, 45 and 60 min during whole‐body heating. The ultrasound probe was placed 5–15 cm proximal to the antecubital fossa, where an optimal B‐mode image could be obtained. A rapid inflation/deflation pneumatic cuff (SC5; Hokanson) was placed immediately distal to the antecubital fossa and inflated to 250 mmHg for 5 min by a rapid cuff inflator (E20; Hokanson). Brachial artery diameter and blood velocity were recorded continuously for 1 min before cuff inflation, during cuff occlusion and for 3 min after cuff deflation. Ultrasound recordings were sent to a remote computer using a frame grabber (DVIUSB 3.0, Epiphan), were video captured and analysed using edge‐detection and wall‐tracking software (Cardiovascular Suite v.3; Quipu SRL). This method provided measurements of arterial diameter and time‐averaged positive (antegrade)/negative (retrograde) blood velocities based on the Doppler envelope, at a sampling rate of 30 Hz.

### 2.6 Data analyses

Baseline brachial artery diameter was defined as the average diameter during the 1 min baseline recording. Peak brachial artery diameter was defined as the greatest 1 s average during the postocclusion period. Flow‐mediated dilatation was determined as the percentage change in brachial artery diameter from baseline to peak. During the whole‐body heating modalities, FMD was also calculated as the percentage change in brachial artery diameter from the last 30 s of occlusion, for comparison with a recent study that took this approach (Coombs et al., 2021). Shear rate was calculated as follows: four times mean blood velocity divided by diameter. Antegrade and retrograde shear rates were calculated using positive and negative mean blood velocity, respectively. The PORH was quantified as peak and total (area under the curve above pre‐occlusion values) forearm vascular conductance (forearm blood flow divided by mean arterial pressure) during the 3 min postocclusion period. All ultrasound recordings were analysed by a single trained researcher who was unblinded to study conditions. Mean skin temperature was expressed as a weighted average of the four measurement sites (Ramanathan, 1964).

### 2.7 Statistical analyses

The sample size was estimated a priori using the only studies available at the time of designing the present study in which an acute improvement in brachial artery FMD in response to heat exposure was observed (Cheng et al., 2019; Tinken et al., 2009). In response to forearm heating with an electric blanket, FMD increased from (mean ± SD) 5.8 ± 2.2% before heating to 8.4 ± 3.6% at ∼5 min post‐heating (Cheng et al., 2019). In response to forearm hot water immersion, FMD increased from 4.6 ± 0.9% before heating to 8.1 ± 5.4% ‘immediately’ post‐heating (Tinken et al., 2009). Based on these findings, the present study was powered to detect an immediate change in FMD of 3 ± 4% (mean ± SD) with a correlation of 0.5 between pre‐ and post‐heating measurements. A sample size of 19 participants was estimated to detect such a change at the 0.05 level with 80% power using a two‐tailed Student's paired *t*‐test.

The primary analyses were performed on the dependent variables expressed as a change from baseline using a mixed‐effects model that included the factors of heating modality (electric blanket, forearm water immersion, whole‐body heating with arm covered and whole‐body heating with arm uncovered) and measurement time (preheating, ≤5, 30, 60, 90 and 120 min post‐heating). To test hypothesis 1 (time course of changes following heating), we considered the main effect of measurement time. Post‐hoc comparisons relative to preheating values were performed with Dunnett's correction. To test hypothesis 2 (magnitude of change between heating modalities), we considered the interaction of time and heating modality. Post‐hoc comparisons between heating modalities were performed with Tukey's correction. To test hypothesis 3 (exposure of limb to heating stimulus), we restricted the analyses to the whole‐body heating modalities (arm covered and uncovered). Post‐hoc comparisons between whole‐body heating modalities were performed with Tukey's correction. To test hypothesis 4 (modulating effect of biological sex), we restricted the analyses to within each heating modality and considered the measurement time × sex interaction of a mixed‐effects model that included the factors of time and biological sex. Post‐hoc comparisons were performed with Fisher's LSD test. We also considered whether sex‐based differences for change in FMD and PORH are equivalent (O'Brien & Kimmerly, 2022). The mean female–male difference for change in FMD or PORH at each measurement time was plotted along with the upper 90% confidence interval and contrasted with an equivalence limit of 3% for FMD, 1 ml/min/mmHg for peak forearm vascular conductance or 1 ml/min/mmHg × min for forearm vascular conductance area under the curve. The equivalence limits were established based on previous studies, in which an acute improvement in brachial artery FMD and/or peak or total PORH was observed in response to limb heating (Cheng et al., 2019, 2021; Romero et al., 2017; Tinken et al., 2009). If the upper 90% confidence interval fell within the equivalence limit, we considered the female–male difference equivalent.

For FMD values, the analyses were performed on unadjusted values and values adjusted for baseline diameter and shear rate area under the curve (SR_AUC_) up to peak diameter (Atkinson, 2014; Cheng et al., 2021). A logarithmic transformation was applied to baseline (ln*D*_base_) and peak (ln*D*_peak_) diameter and SR_AUC_ (lnSR_AUC_). These adjustments were performed because FMD expressed as a percentage change was negatively correlated with baseline brachial artery diameter (*r* ranged from −0.30 to −0.70, all *P* < 0.01) and positively with SR_AUC_ (*r* ranged from 0.42 to 0.55, all *P* < 0.01) within each heating modality. The change in diameter on the logarithmic scale (ln*D*_diff_ = ln*D*_peak_ − ln*D*_base_) was used as the dependent variable in a mixed‐effects model, with measurement time as a fixed factor and ln*D*_base_ and lnSR_AUC_ as covariates. Covariate‐adjusted estimated means (EM) and standard errors for ln*D*_diff_ were obtained from each model at each time point for each heating modality and were then back‐transformed to provide the mean scaled FMD values as [(e^EM^ − 1) × 100] and the standard deviation as [((e^SE^ − 1) × 100) × √𝑛]; where *n* is the sample size.

To characterize the physiological changes elicited by the heating modalities, dependent variables were analysed as the change from preheating using a mixed‐effects model that included the factors of heating modality and measurement time (preheating, end of heating, and ≤5, 30, 60, 90 and 120 min post‐heating). Descriptive results are presented as the mean ± SD. Differences are presented as the mean with 95% confidence interval. Statistical significance was set at an α‐level of ≤0.05. Statistical analyses were performed using commercially available software (Prism v.8.0.1; GraphPad Software, La Jolla, CA, USA).

## 3 RESULTS

Of the 20 participants recruited for this study, two female participants did not complete all visits owing to the required time commitment. One completed two visits (forearm water immersion and whole‐body heating with arm covered), and the other completed one visit (forearm water immersion). This resulted in a final sample size of *n* = 18 for electric blanket heating (11 males and seven females), *n* = 20 for forearm hot water immersion (11 males and nine females), *n* = 19 for whole‐body heating with the arm covered (11 males and eight females) and *n* = 18 for whole‐body heating with the arm uncovered (11 males and seven females).

### 3.1 Physiological responses during and after heat exposure

**FIGURE 1:**
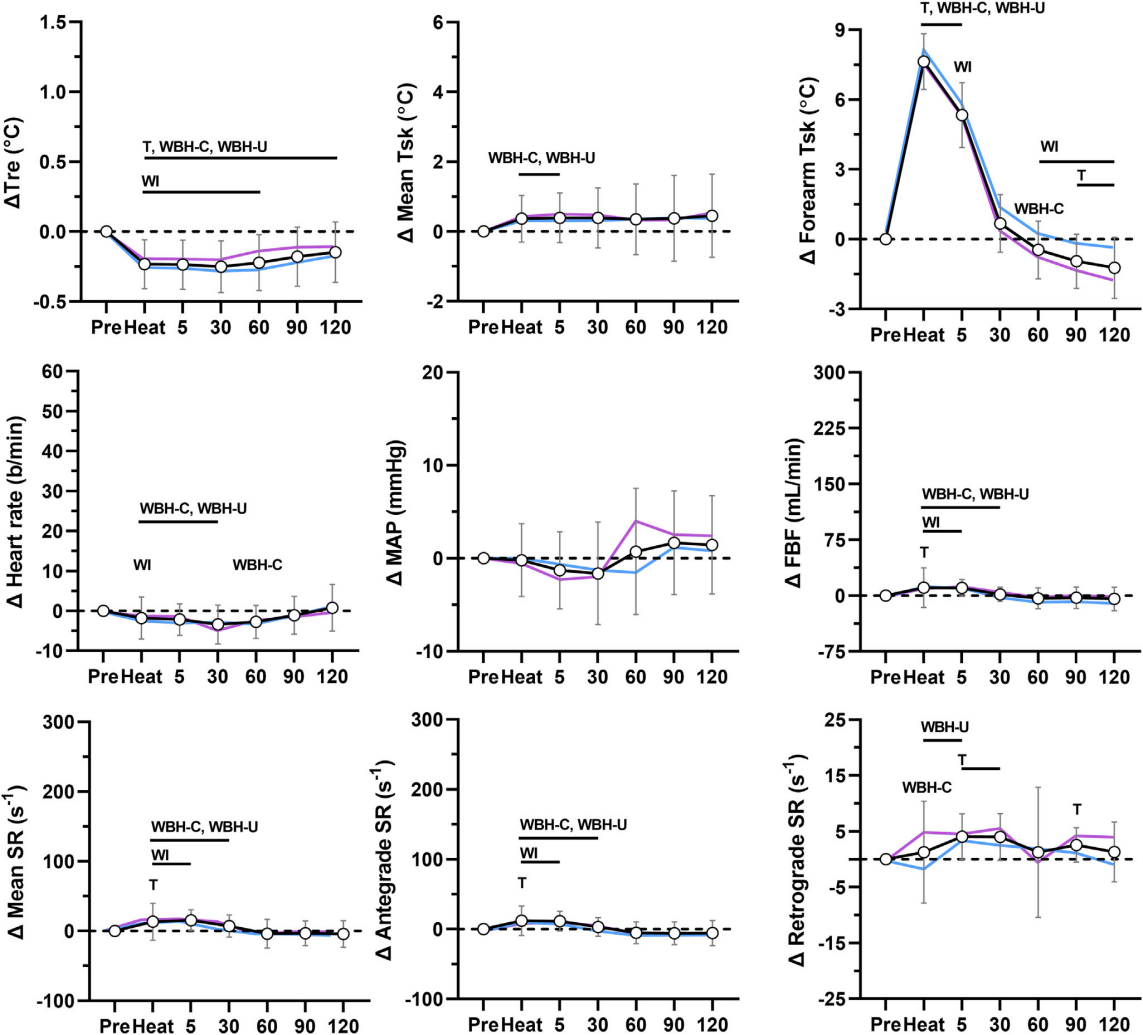
Physiological responses during forearm heating with an electric blanket. The data for the combined group of males and females (*n* = 18) are presented as the mean ± SD (black lines with open circles). The mean values for males (*n* = 11, blue) and females (*n* = 7, pink) are overlaid without SD values for clarity. Data are presented before (Pre), during (Heat) and after 10 min of heating the forearm with an electric blanket. Top panels: change (Δ) in rectal (Tre), mean skin (Mean Tsk) and forearm skin (Forearm Tsk) temperatures. Middle panels: change in heart rate, mean arterial pressure (MAP) and forearm blood flow (FBF). Bottom panels: change in mean, antegrade and retrograde shear rate (SR). T, *P* ≤ 0.05 for post‐hoc comparison versus Pre (Dunnett's correction). WI, *P* ≤ 0.05 for post‐hoc comparison versus forearm water immersion (Tukey's correction). WBH‐C, *P* ≤ 0.05 for post‐hoc comparison versus whole‐body heating with arm covered (Tukey's correction). WBH‐U, *P* ≤ 0.05 for post‐hoc comparison versus whole‐body heating with arm uncovered (Tukey's correction)

**FIGURE 2:**
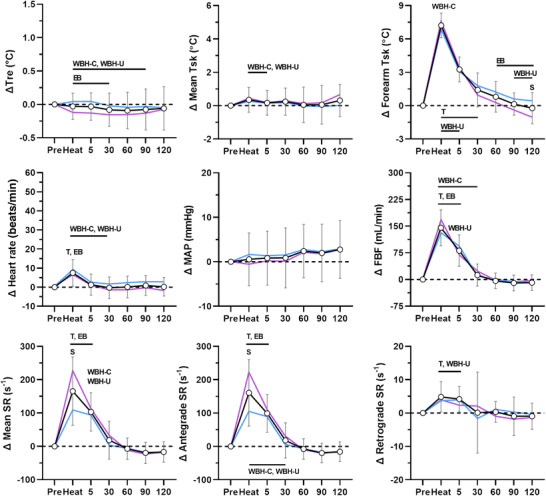
Physiological responses during forearm hot water immersion. The data for the combined group of males and females (*n* = 20) are presented as the mean ± SD (black lines with open circles). The mean values for males (*n* = 11, blue) and females (*n* = 9, pink) are overlaid without SD values for clarity. Data are presented before (Pre), during (Heat) and after 30 min of heating the forearm in 42°C water. Top panels: change (Δ) in rectal (Tre), mean skin (Mean Tsk) and forearm skin (Forearm Tsk) temperatures. Middle panels: change in heart rate, mean arterial pressure (MAP) and forearm blood flow (FBF). Bottom panels: change in mean, antegrade and retrograde shear rate (SR). T, *P* ≤ 0.05 for post‐hoc comparison versus Pre (Dunnett's correction). EB, *P* ≤ 0.05 for post‐hoc comparison versus electric blanket (Tukey's correction). WBH‐C, *P* ≤ 0.05 for post‐hoc comparison versus whole‐body heating with arm covered (Tukey's correction). WBH‐U, *P* ≤ 0.05 for post‐hoc comparison versus whole‐body heating with arm uncovered (Tukey's correction). S, *P* ≤ 0.05 for post‐hoc comparison between males and females (Fisher's LSD test)

**FIGURE 3:**
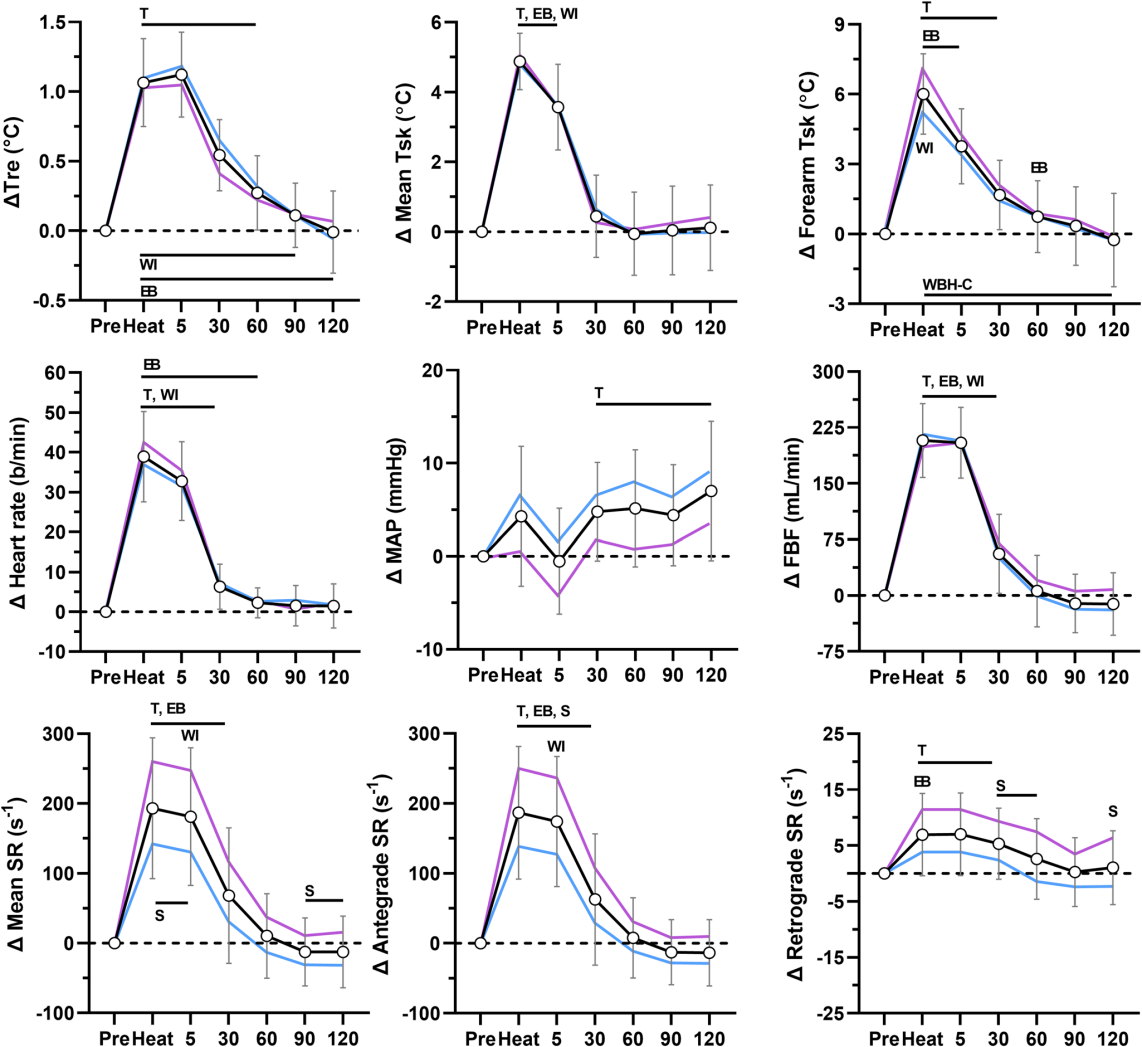
Physiological responses during whole‐body heating with arm covered. The data for the combined group of males and females (*n* = 19) are presented as the mean ± SD (black lines with open circles). The mean values for males (*n* = 11, blue) and females (*n* = 8, pink) are overlaid without SD values for clarity. Data are presented before (Pre), during (Heat) and after 60 min of whole‐body passive heating with the arm exposed to the heating stimulus. Top panels: change (Δ) in rectal (Tre), mean skin (Mean Tsk) and forearm skin (Forearm Tsk) temperatures. Middle panels: change in heart rate, mean arterial pressure (MAP) and forearm blood flow (FBF). Bottom panels: change in mean, antegrade and retrograde shear rate (SR). T, *P* ≤ 0.05 for post‐hoc comparison versus Pre (Dunnett's correction). EB, *P* ≤ 0.05 for post‐hoc comparison versus electric blanket (Tukey's correction). WI, *P* ≤ 0.05 for post‐hoc comparison versus forearm water immersion (Tukey's correction). WBH‐U, *P* ≤ 0.05 for post‐hoc comparison versus whole‐body heating with arm uncovered (Tukey's correction). S, *P* ≤ 0.05 for post‐hoc comparison between males and females (Fisher's LSD test)

**FIGURE 4:**
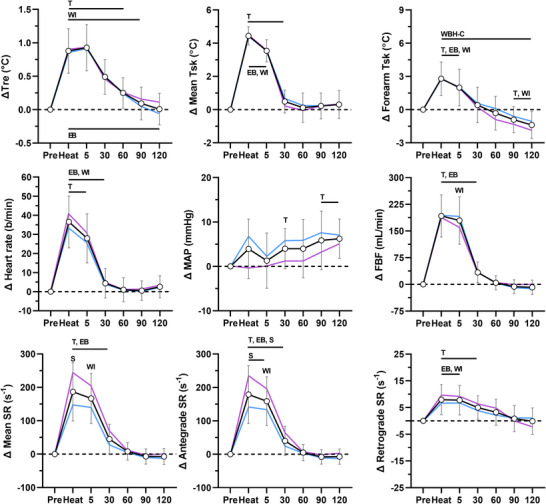
Physiological responses during whole‐body heating with arm uncovered. The data for the combined group of males and females (*n* = 18) are presented as the mean ± SD (black lines with open circles). The mean values for males (*n* = 11, blue) and females (*n* = 7, pink) are overlaid without SD values for clarity. Data are presented before (Pre), during (Heat) and after 60 min of whole‐body passive heating with the arm not exposed to the heating stimulus. Top panels: change (Δ) in rectal (Tre), mean skin (Mean Tsk) and forearm skin (Forearm Tsk) temperatures. Middle panels: change in heart rate, mean arterial pressure (MAP) and forearm blood flow (FBF). Bottom panels: change in mean, antegrade and retrograde shear rate (SR). T, *P* ≤ 0.05 for post‐hoc comparison versus Pre (Dunnett's correction). EB, *P* ≤ 0.05 for post‐hoc comparison versus electric blanket (Tukey's correction). WI, *P* ≤ 0.05 for post‐hoc comparison versus forearm water immersion (Tukey's correction). WBH‐C, *P* ≤ 0.05 for post‐hoc comparison versus whole‐body heating with arm covered (Tukey's correction). S, *P* ≤ 0.05 for post‐hoc comparison between males and females (Fisher's LSD test)

The physiological responses during each heating modality are presented in Figures 1, 2, 3, 4. Rectal and mean skin temperatures remained relatively stable during limb heating, whereas they increased during whole‐body heating (interactions, *P* < 0.001). The change in rectal (*P* = 0.129) and mean skin (*P* = 0.101) temperatures at the end of whole‐body heating did not differ between the arm covered and uncovered modalities. After whole‐body heating, mean skin and rectal temperatures remained above preheating values until vascular function measurements performed at 30 and 60 min, respectively. The change in rectal (interactions, *P* ≥ 0.073) and mean skin (interactions, *P* ≥ 0.570) temperatures did not differ between males and females for each heating modality. Forearm skin temperature increased from preheating values during limb and whole‐body heating (effect of time, *P* < 0.001). The change in forearm skin temperature during heat exposure was greater during limb heating compared with whole‐body heating (all *P* < 0.044). During limb heating, the change in forearm skin temperature did not differ between the electric blanket and hot water immersion (*P* = 0.733). During whole‐body heating, the change in forearm skin temperature was greater when the arm was covered versus uncovered (*P* < 0.001). There was a sex × time interaction for the change in forearm skin temperature during forearm water immersion (*P* < 0.001), because it remained at greater values above preheating in males during the post‐heating period. There were no sex‐based differences in forearm skin temperature during the other heating modalities (interactions, *P* ≥ 0.061).

Heart rate increased during whole‐body heating, but not limb heating (interaction, *P* < 0.001). Mean arterial pressure increased over the course of the protocol (effect of time, *P* < 0.001), but this increase did not differ between heating modalities (interaction, *P* = 0.184). There were no differences between males and females for the change in heart rate (interactions, *P* ≥ 0.542) and mean arterial pressure (interactions, *P* ≥ 0.177). Forearm blood flow increased during all heating modalities (effect of time, *P* < 0.001). During limb heating, the change in forearm blood flow was greater during water immersion compared with the electric blanket (*P* < 0.001). During whole‐body heating, the change in forearm blood flow did not differ between the arm covered versus uncovered modalities (*P* = 0.592). Whole‐body heating with the arm covered (*P* = 0.022), but not uncovered (*P* = 0.244), elicited a greater change in forearm blood flow relative to forearm water immersion. The change in forearm blood flow did not differ between males and females during each heating modality (interactions, *P* ≥ 0.150).

Mean shear rate increased during heating (effect of time, *P* < 0.001). During limb heating, the increase in mean shear rate was greater during hot water immersion compared with the electric blanket (*P* = 0.001). During whole‐body heating, the increase in mean shear rate did not differ between the arm covered and uncovered modalities (*P* = 0.979). The change in mean shear rate did not differ between forearm water immersion and both whole‐body heating modalities (*P* ≥ 0.635). The increase in mean shear rate during heating was mediated by an increase in antegrade shear rate (*P* < 0.001) and decrease in retrograde shear rate (*P* < 0.001). Shear rate returned to preheating values within 60 min after all heating modalities. During electric blanket heating, there were no differences between males and females for the change in mean (*P* = 0.953), antegrade (*P* = 0.969) and retrograde (*P* = 0.298) shear rate. During forearm water immersion, the change in mean (*P* = 0.003) and antegrade (*P* = 0.002) shear rate differed between males and females. A greater change was observed in females at the end of heating (post‐hoc comparisons, *P* ≤ 0.050). The change in retrograde shear rate did not differ between males and females (*P* = 0.313). During whole‐body heating with the arm covered and uncovered, the change in mean (interactions, *P* ≤ 0.002), antegrade (interactions, *P* ≤ 0.004) and retrograde (interactions, *P* ≤ 0.010) shear rate differed between males and females. A greater change in mean and antegrade shear rate was observed in females at the end of both whole‐body heating modalities (post‐hoc comparisons, *P* ≤ 0.029). Retrograde shear rate generally remained at greater values above preheating values (less negative) in females during the post‐heating period (post‐hoc comparisons, *P* ≤ 0.039).

### 3.2 Objective 1: What is the time course of acute changes in brachial artery FMD and PORH following limb and whole‐body heating?

**FIGURE 5:**
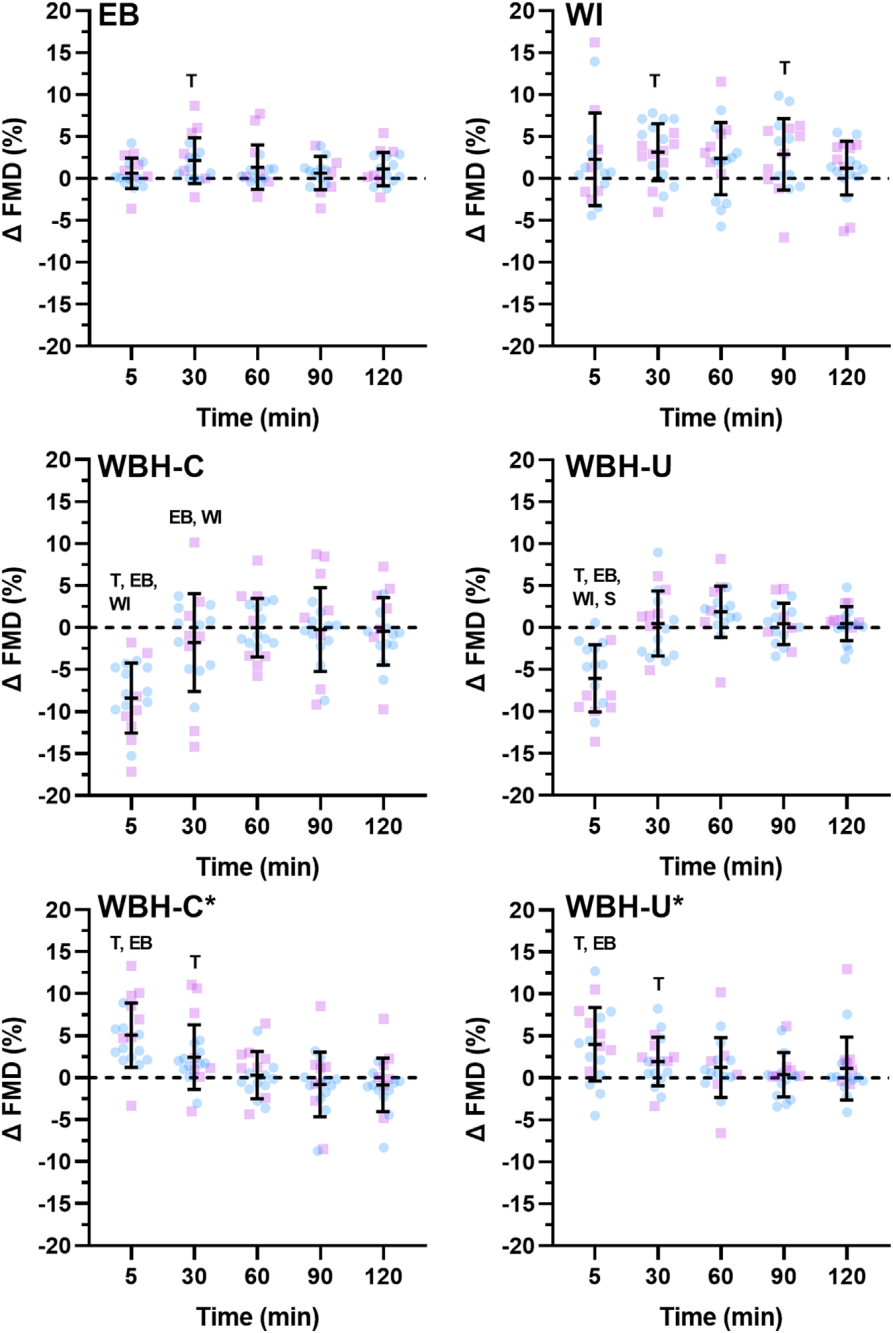
Brachial artery flow‐mediated dilatation (FMD) following limb and whole‐body heating modalities. Data are presented as the mean ± SD with individual values for males (blue) and females (pink) following forearm heating with an electric blanket (EB, *n* = 18), forearm hot water immersion (WI, *n* = 20), whole‐body heating with the arm covered (WBH‐C, *n* = 19), whole‐body heating with the arm uncovered (WBH‐U, *n* = 18) and whole‐body heating with the arm covered and uncovered when FMD was calculated from end‐occlusion diameter (WBH‐C* and WBH‐U*). T, *P* ≤ 0.05 for post‐hoc comparison versus Pre (Dunnett's correction). EB, *P* ≤ 0.05 for post‐hoc comparison versus electric blanket (Tukey's correction). WI, *P* ≤ 0.05 for post‐hoc comparison versus forearm water immersion (Tukey's correction). S, *P* ≤ 0.05 for post‐hoc comparison between males and females (Fisher's LSD test)

**FIGURE 6:**
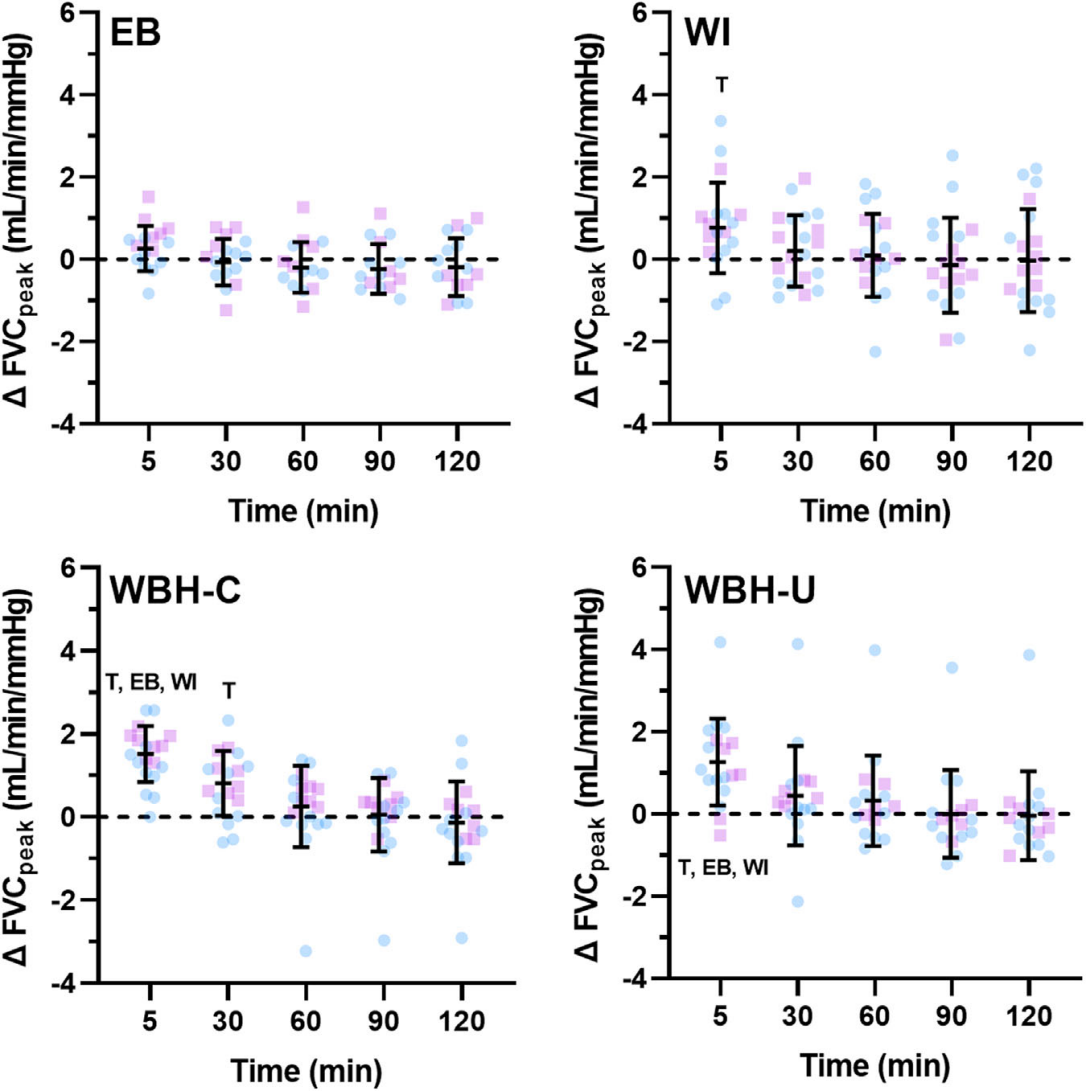
Peak forearm vascular conductance (FVC_peak_) during reactive hyperaemia following limb and whole‐body heating modalities. Data are presented as the mean ± SD with individual values for males (blue) and females (pink) following forearm heating with an electric blanket (EB, *n* = 18), forearm hot water immersion (WI, *n* = 20), whole‐body heating with the arm covered (WBH‐C, *n* = 19) and whole‐body heating with the arm uncovered (WBH‐U, *n* = 18). T, *P* ≤ 0.05 for post‐hoc comparison versus Pre (Dunnett's correction). EB, *P* ≤ 0.05 for post‐hoc comparison versus electric blanket (Tukey's correction). WI, *P* ≤ 0.05 for post‐hoc comparison versus forearm water immersion (Tukey's correction)

**FIGURE 7:**
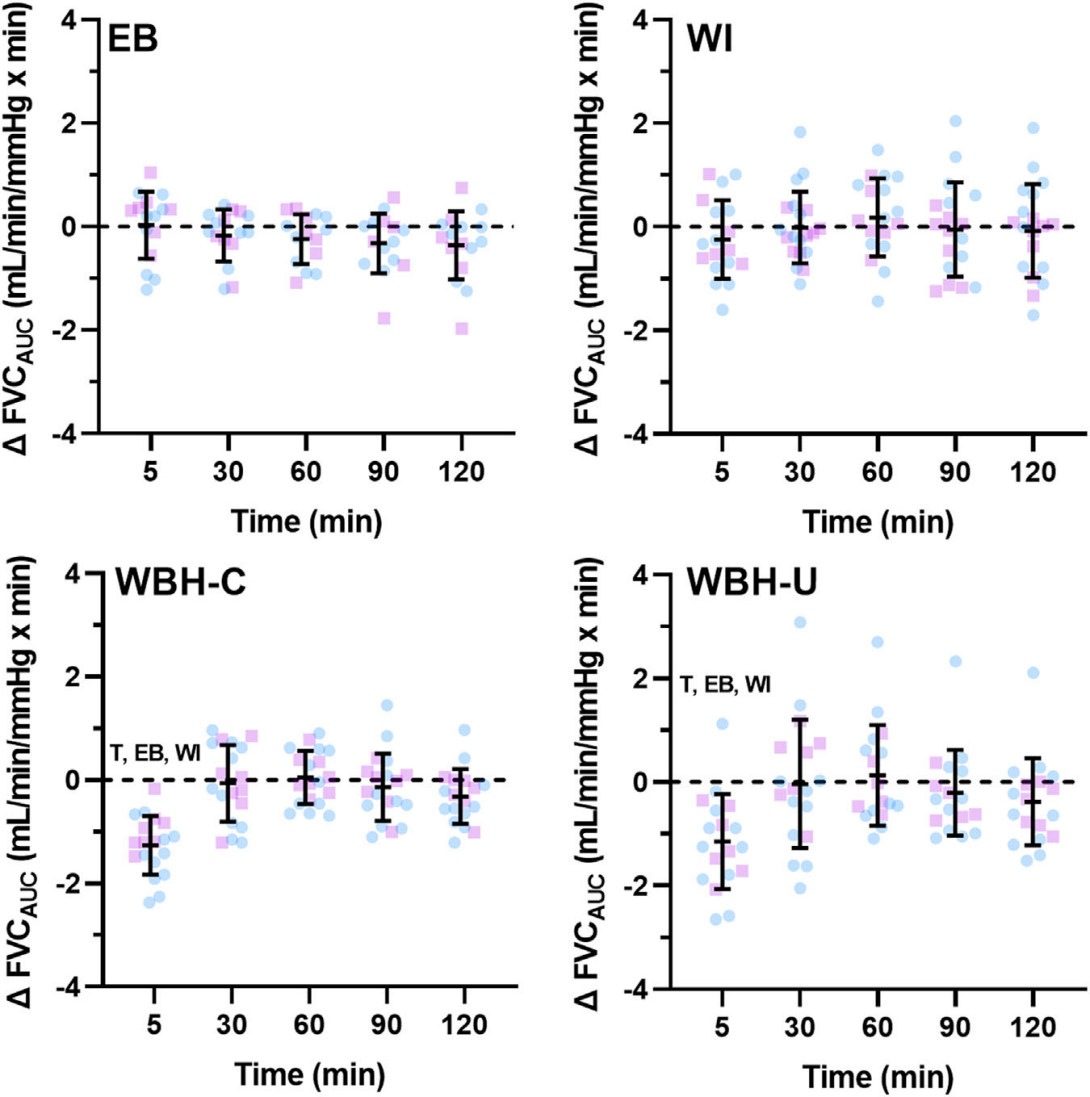
Forearm vascular conductance area under the curve (FVC_AUC_) during reactive hyperaemia following limb and whole‐body heating modalities. Data are presented as the mean ± SD with individual values for males (blue) and females (pink) following forearm heating with an electric blanket (EB, *n* = 18), forearm hot water immersion (WI, *n* = 20), whole‐body heating with the arm covered (WBH‐C, *n* = 19) and whole‐body heating with the arm uncovered (WBH‐U, *n* = 18). T, *P* ≤ 0.05 for post‐hoc comparison versus Pre (Dunnett's correction). EB, *P* ≤ 0.05 for post‐hoc comparison versus electric blanket (Tukey's correction). WI, *P* ≤ 0.05 for post‐hoc comparison versus forearm water immersion (Tukey's correction)

Mean brachial artery FMD and PORH values are presented in Table 1, and individual values are presented in Figures 5, 6, 7. Baseline brachial artery diameter (*P* < 0.001) and SR_AUC_ to peak diameter (*P* < 0.001) changed over time during the protocol. Baseline diameter was greater than preheating values ≤5 min following forearm water immersion (*P* = 0.019), and at ≤5 (both *P* < 0.001) and 30 min (both *P* ≤ 0.035) after both whole‐body heating modalities. The SR_AUC_ to peak diameter increased from preheating values 60 min following forearm water immersion (*P* = 0.045), whereas it decreased ≤5 min following whole‐body heating with the arm covered (*P* = 0.004).

**TABLE 1:** Mean values during brachial artery vascular function measurements

**Parameter**	**Baseline**	**5 min**	**30 min**	**60 min**	**90 min**	**120 min**
***D*_base_ (mm)**						
EB	3.78 ± 0.56	3.83 ± 0.46	3.75 ± 0.50	3.70 ± 0.45	3.82 ± 0.43	3.78 ± 0.47
WI	3.64 ± 0.52	3.93 ± 0.55*	3.72 ± 0.47	3.71 ± 0.51	3.59 ± 0.49	3.67 ± 0.47
WBH‐C	3.67 ± 0.67	4.49 ± 0.57*	4.02 ± 0.62*	3.77 ± 0.61	3.73 ± 0.64	3.70 ± 0.68
WBH‐U	3.79 ± 0.56	4.42 ± 0.52*	3.93 ± 0.62*	3.82 ± 0.56	3.85 ± 0.57	3.73 ± 0.60
***D*_occ_ (mm)**						
EB	3.78 ± 0.60	3.79 ± 0.44	3.74 ± 0.47	3.70 ± 0.44	3.82 ± 0.45	3.77 ± 0.46
WI	3.64 ± 0.53	3.82 ± 0.53	3.75 ± 0.50	3.75 ± 0.50	3.62 ± 0.51	3.69 ± 0.51
WBH‐C	3.65 ± 0.66	3.94 ± 0.60*	3.85 ± 0.65*	3.75 ± 0.64	3.73 ± 0.63	3.70 ± 0.67
WBH‐U	3.78 ± 0.57	4.01 ± 0.58*	3.87 ± 0.58	3.84 ± 0.56	3.84 ± 0.60	3.70 ± 0.61
***D*_peak_ (mm)**						
EB	3.92 ± 0.55	4.00 ± 0.46*	3.95 ± 0.46	3.88 ± 0.39	3.99 ± 0.42	3.95 ± 0.43
WI	3.89 ± 0.53	4.28 ± 0.48	4.09 ± 0.48	4.03 ± 0.45	3.95 ± 0.53	3.96 ± 0.45
WBH‐C	3.88 ± 0.61	4.39 ± 0.53*	4.19 ± 0.58*	4.00 ± 0.58	3.94 ± 0.60	3.91 ± 0.60
WBH‐U	3.95 ± 0.55	4.35 ± 0.54*	4.12 ± 0.59*	4.06 ± 0.54*	4.03 ± 0.54	3.90 ± 0.57
**FMD (mm)**						
EB	0.14 ± 0.07	0.17 ± 0.07	0.21 ± 0.10*	0.17 ± 0.10	0.17 ± 0.08	0.17 ± 0.09
WI	0.25 ± 0.10	0.35 ± 0.15	0.37 ± 0.11*	0.32 ± 0.14	0.36 ± 0.17*	0.29 ± 0.12
WBH‐C	0.21 ± 0.13	−0.10 ± 0.12*	0.17 ± 0.16	0.23 ± 0.10	0.21 ± 0.12	0.21 ± 0.12
WBH‐U	0.16 ± 0.09	−0.07 ± 0.14*	0.18 ± 0.13	0.24 ± 0.11*	0.18 ± 0.11	0.17 ± 0.09
**FMD (%)**						
EB	3.93 ± 1.94	4.43 ± 2.00	5.85 ± 3.35*	4.98 ± 3.24	4.55 ± 2.46	4.80 ± 2.84
WI	6.96 ± 2.78	9.44 ± 5.37	10.10 ± 3.30*	9.05 ± 4.53	10.19 ± 4.69*	8.15 ± 3.68
WBH‐C	6.23 ± 4.31	−2.05 ± 2.41*	4.58 ± 4.35	6.35 ± 3.19	6.10 ± 3.96	6.12 ± 3.89
WBH‐U	4.41 ± 2.48	−1.64 ± 3.33*	4.94 ± 3.56	6.50 ± 3.36*	4.88 ± 3.50	4.92 ± 2.76
**SR_AUC_ × 10^3^ (s^−1^ × s)**						
EB	8.4 ± 2.0	8.2 ± 1.9	8.0 ± 2.6	8.2 ± 2.5	7.1 ± 2.4	7.5 ± 2.4
WI	8.1 ± 2.1	7.6 ± 3.3	8.3 ± 2.5	9.4 ± 3.0*	8.4 ± 3.1	8.8 ± 2.6
WBH‐C	8.9 ± 2.9	6.2 ± 3.0*	9.0 ± 2.9	8.9 ± 3.1	8.0 ± 2.6	8.0 ± 2.8
WBH‐U	8.2 ± 2.8	5.2 ± 2.7*	8.6 ± 4.0	8.3 ± 3.2	7.6 ± 2.3	7.3 ± 2.2
**FVC_base_ (ml/min/mmHg)**						
EB	0.35 ± 0.22	0.47 ± 0.24*	0.38 ± 0.18	0.30 ± 0.16	0.32 ± 0.15	0.29 ± 0.13
WI	0.41 ± 0.27	1.41 ± 0.57*	0.55 ± 0.38	0.35 ± 0.19	0.26 ± 0.19	0.28 ± 0.17
WBH‐C	0.37 ± 0.27	3.20 ± 0.81*	1.19 ± 0.68*	0.57 ± 0.30*	0.37 ± 0.16	0.36 ± 0.19
WBH‐U	0.42 ± 0.33	2.68 ± 0.93*	0.80 ± 0.42*	0.45 ± 0.26*	0.33 ± 0.13	0.29 ± 0.12
**FVC_peak_ (ml/min/mmHg)**						
EB	2.78 ± 0.80	3.21 ± 0.75*	2.85 ± 0.80	2.74 ± 0.87	2.78 ± 0.84	2.81 ± 0.93
WI	2.92 ± 1.04	3.86 ± 0.83*	3.19 ± 1.04	3.14 ± 0.90	2.64 ± 0.93	2.95 ± 1.04
WBH‐C	2.81 ± 1.20	4.28 ± 1.15*	3.57 ± 0.93*	3.02 ± 0.88	2.81 ± 0.88	2.67 ± 0.95
WBH‐U	2.68 ± 1.06	3.95 ± 1.21*	3.14 ± 0.93	3.04 ± 0.99	2.78 ± 0.87	2.65 ± 0.97
**FVC_AUC_ (ml/min/mmHg × min)**						
EB	1.66 ± 0.69	1.79 ± 0.58	1.55 ± 0.65	1.49 ± 0.53	1.44 ± 0.53	1.45 ± 0.71
WI	1.55 ± 0.61	1.39 ± 0.40	1.55 ± 0.57	1.68 ± 0.53	1.46 ± 0.58	1.47 ± 0.57
WBH‐C	1.69 ± 0.57	0.42 ± 0.36*	1.62 ± 0.72	1.73 ± 0.75	1.54 ± 0.74	1.39 ± 0.60
WBH‐U	1.66 ± 0.79	0.49 ± 0.45*	1.60 ± 0.74	1.81 ± 0.78	1.48 ± 0.62	1.25 ± 0.64

Values are presented as the mean ± SD.

Abbreviations: *D*_base_, baseline diameter; *D*_occ_, end‐occlusion diameter; *D*_peak_, peak diameter; EB, electric blanket; FMD; flow‐mediated dilatation; FVC_AUC_, forearm vascular conductance area under the curve during reactive hyperaemia; FVC_base_, pre‐occlusion forearm vascular conductance; FVC_peak_, peak forearm vascular conductance during reactive hyperaemia; SR_AUC_, shear rate area under the curve to peak diameter; WBH‐C, whole‐body heating with arm covered; WBH‐U, whole‐body heating with arm uncovered; WI, forearm hot water immersion.

* *P* ≤ 0.05 for post‐hoc comparison versus baseline (Dunnett's correction following main effect of time with a mixed‐effects model).

There was a main effect of time for change in brachial artery FMD (*P* < 0.001). In response to limb heating, FMD increased from baseline values 30 min after electric blanket heating (2.15% [0.29, 4.01], *P* = 0.021), and at 30 (3.14% [1.04, 5.24], *P* = 0.003) and 90 min (2.89% [0.15, 5.62], *P* = 0.037) after hot water immersion. In response to whole‐body heating, FMD calculated using pre‐occlusion diameter decreased 5 min post‐heating when the arm was covered (−8.23% [−10.93, −5.64], *P* < 0.001) and uncovered (−6.04% [−8.68, −3.41], *P* < 0.001). When calculated using end‐occlusion diameter, FMD increased from baseline values ≤5 and 30 min post‐heating when the arm was covered (≤5 min, 5.19% [2.76, 7.62], *P* < 0.001; and 30 min, 2.56% [0.12, 4.99], *P* = 0.038) and uncovered (≤5 min, 4.03% [1.16, 6.91], *P* = 0.005; and 30 min, 1.99% [0.09, 3.90], *P* = 0.039). The increase in FMD when calculated from end‐occlusion values, as opposed to the decrease observed when calculated from pre‐occlusion values, was paralleled by a decrease in brachial artery diameter during occlusion at ≤5 (covered, −0.53 ± 0.16 mm; and uncovered, −0.40 ± 0.21 mm) and 30 min (covered, −0.15 ± 0.20 mm; and uncovered, −0.06 ± 0.12 mm) after both whole‐body heating modalities. When adjusting FMD for baseline diameter and SR_AUC_ to peak diameter, a main effect of time was still observed during each heating modality (electric blanket, *P* = 0.038; water immersion, *P* = 0.042; whole‐body heating with arm covered, *P* < 0.001; and whole‐body heating with arm uncovered, *P* < 0.001).

Pre‐occlusion peak and total forearm vascular conductance changed over time during the protocol (all *P* < 0.001). Pre‐occlusion forearm vascular conductance increased from baseline ≤5 min following both limb heating modalities (both *P* ≤ 0.002), at ≤5, 30 and 60 min after whole‐body heating with the arm covered (all *P* ≤ 0.028) and ≤5 and 30 min after whole‐body heating with the arm uncovered (both *P* < 0.001). Peak forearm vascular conductance during PORH increased from baseline values ≤5 min following forearm water immersion and following whole‐body heating with the arm covered and uncovered (all *P* ≤ 0.025). Peak forearm vascular conductance also increased 30 min following whole‐body heating with the arm covered (*P* = 0.002). Total forearm vascular conductance during PORH decreased ≤5 min following both whole‐body heating modalities (both *P* < 0.001).

### 3.3 Objective 2: Are there differences in the acute effect of heat exposure on brachial artery FMD and PORH between limb and whole‐body heating?

There was a measurement time × modality interaction for change in FMD when it was calculated from pre‐occlusion diameter and when it was calculated from end‐occlusion diameter during the whole‐body heating modalities (both *P* < 0.001). When FMD was calculated as the change from pre‐occlusion diameter, the interaction was driven by the decrease in brachial artery FMD ≤5 min following both whole‐body heating modalities. Consequently, FMD was different between whole‐body and limb heating modalities ≤5 min post‐heating (all comparisons, *P* < 0.001). The change in FMD 30 min post‐heating also differed between whole‐body heating with the arm covered compared with forearm water immersion (*P* = 0.035) and forearm heating with the electric blanket (*P* = 0.017). When FMD was calculated from end‐occlusion diameter, the change in FMD was greater ≤5 min following both whole‐body heating modalities compared with electric blanket heating (both *P* ≤ 0.028). There were also time × modality interactions for pre‐occlusion forearm vascular conductance (*P* < 0.001) and for peak (*P* = 0.032) and total (*P* < 0.001) forearm vascular conductance during PORH. These interactions were driven by greater changes ≤5 min after both whole‐body heating modalities.

### 3.4 Objective 3: Is exposure of the limb from which FMD and PORH are measured required to observe an acute improvement in brachial artery FMD following whole‐body heating?

The change in FMD did not differ between whole‐body heating with the arm covered versus uncovered when FMD was calculated from pre‐occlusion (interaction, *P* = 0.405) or end‐occlusion (*P* = 0.057) diameter. There was a measurement time × modality interaction for pre‐occlusion forearm vascular conductance, because the change ≤5 min post‐heating was greater following whole‐body heating with the arm covered versus uncovered (*P* = 0.022). There were no time × modality interactions for the change in peak (*P* = 0.386) or total (*P* = 0.871) forearm vascular conductance during PORH.

### 3.5 Objective 4: Does biological sex modulate the acute effect of heat exposure on brachial artery FMD and PORH?

When FMD was calculated using pre‐occlusion diameter, there were no sex × time interactions during forearm water immersion (*P* = 0.105), electric blanket heating (*P* = 0.154) and whole‐body heating with the arm covered (*P* = 0.194). There was a sex × time interaction during whole‐body heating with the arm uncovered (*P* = 0.011), because a greater decrease in brachial artery FMD was observed in females 5 min post‐heating (*P* = 0.032). When FMD was calculated using end‐occlusion diameter, there were no sex × time interactions in response to both whole‐body heating modalities (*P* ≥ 0.423). There were sex × time interactions for the change in pre‐occlusion brachial artery diameter during electric blanket heating (*P* = 0.006) and whole‐body heating with the arm covered (*P* = 0.032). During electric blanket heating, the change in diameter was greater in females ≤5 (*P* = 0.011), 60 (*P* = 0.017), 90 (*P* < 0.001) and 120 min (*P* = 0.004) post‐heating. During whole‐body heating with the arm covered, the change in diameter was greater in females ≤5 min post‐heating (*P* = 0.023). There were no sex × time interactions for SR_AUC_ to peak diameter (*P* ≥ 0.481), pre‐occlusion forearm vascular conductance (*P* ≥ 0.382), peak forearm vascular conductance (*P* ≥ 0.178) or total forearm vascular conductance (*P* ≥ 0.117) during each heating modality.

**FIGURE 8:**
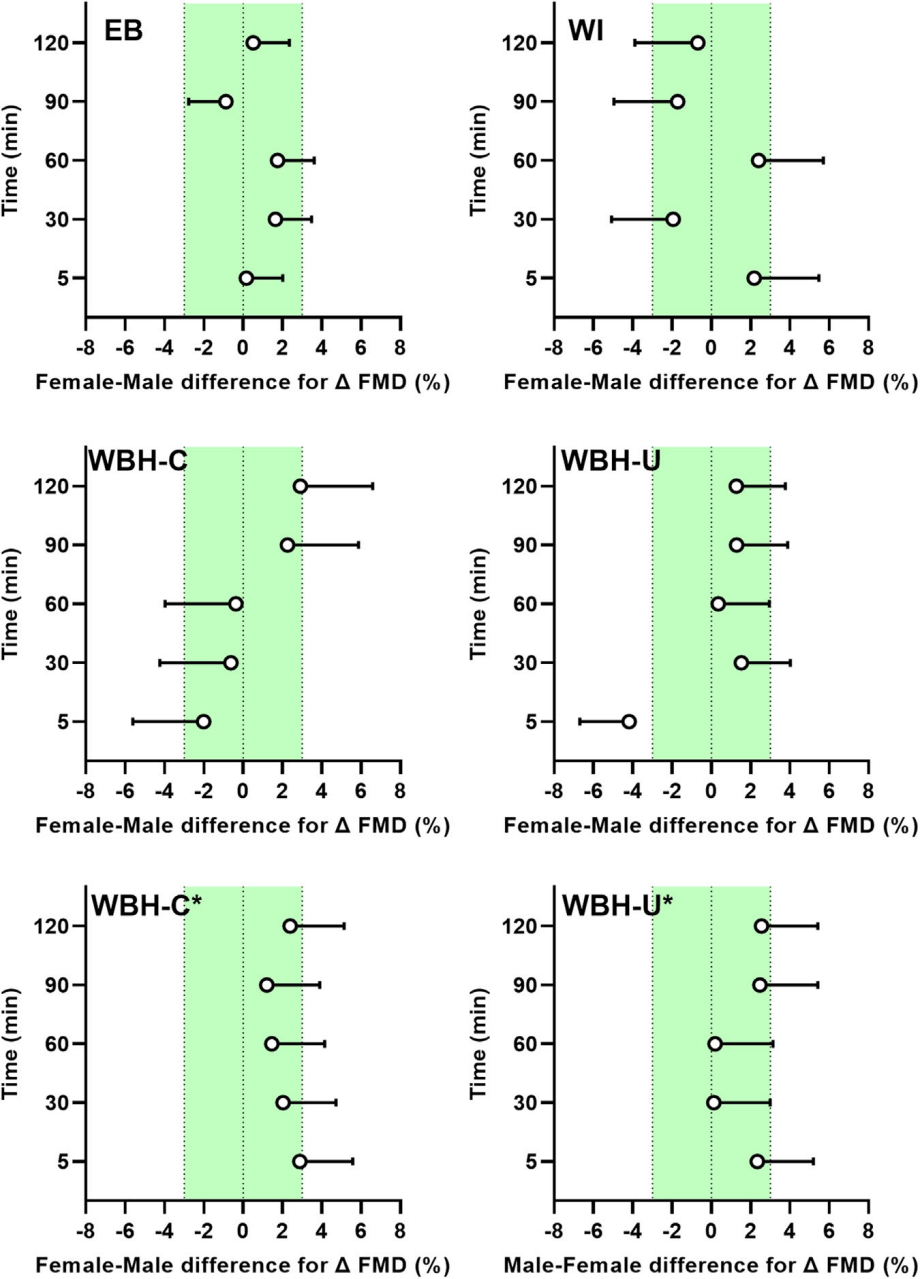
Female–male difference for change in brachial artery flow‐mediated dilatation (FMD) in response to limb and whole‐body heating modalities. Data are presented as the mean difference (females minus males) with upper 90% confidence interval after forearm heating with an electric blanket (EB, 11 males and seven females), forearm hot water immersion (WI, 11 males and nine females), whole‐body heating with the arm covered (WBH‐C, 11 males and eight females), whole‐body heating with the arm uncovered (WBH‐U, 11 males and seven females) and whole‐body heating with the arm covered and uncovered when FMD was calculated from end‐occlusion diameter (WBH‐C* and WBH‐U*). The green shading depicts the equivalence limit (3%)

**FIGURE 9:**
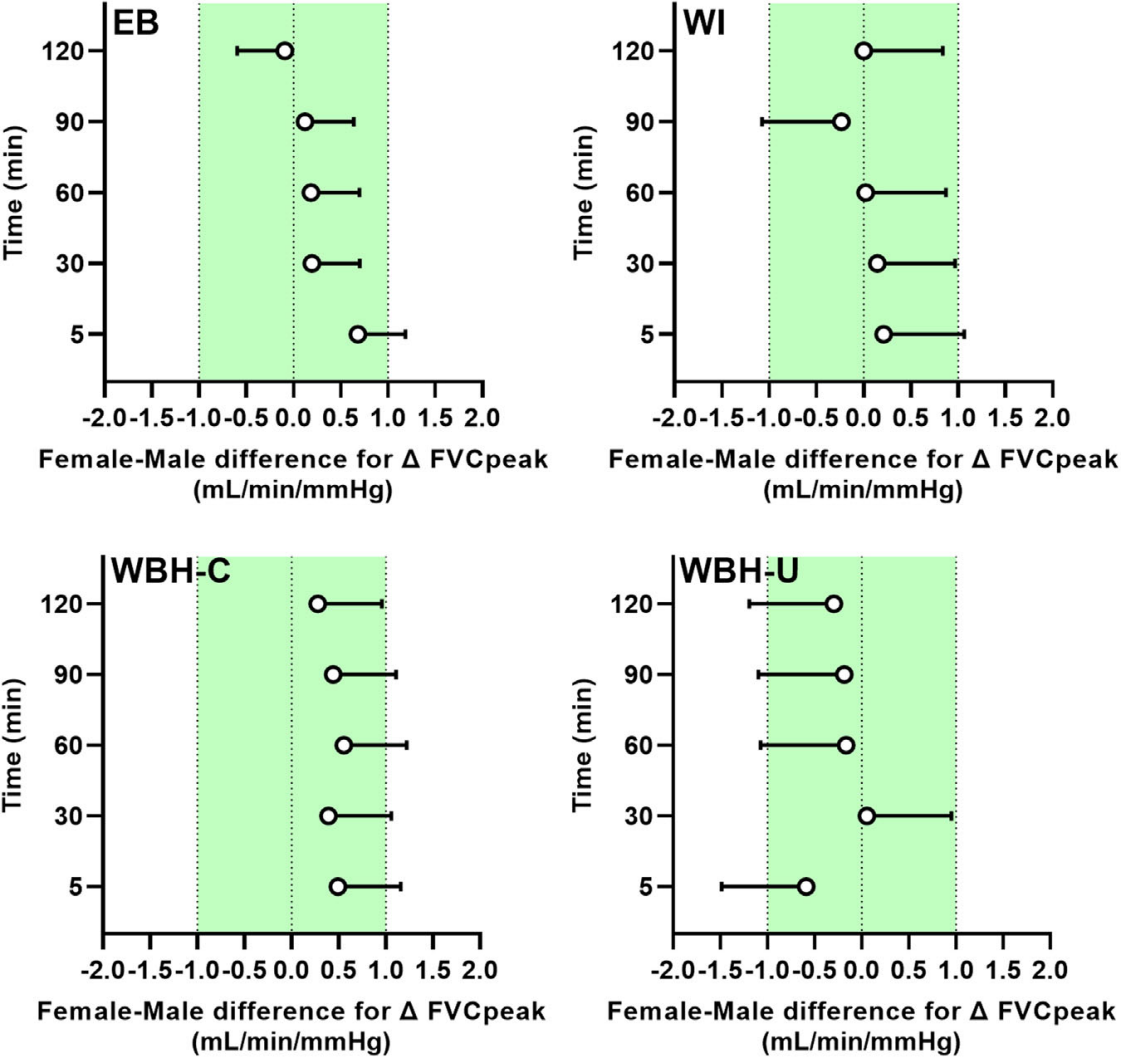
Female–male difference for change in peak forearm vascular conductance (FVC_peak_) during reactive hyperaemia in response to limb and whole‐body heating modalities. Data are presented as the mean difference (females minus males) with upper 90% confidence interval after forearm heating with an electric blanket (EB, 11 males and seven females), forearm hot water immersion (WI, 11 males and nine females), whole‐body heating with the arm covered (WBH‐C, 11 males and eight females) and whole‐body heating with the arm uncovered (WBH‐U, 11 males and seven females). The green shading depicts the equivalence limit (1 ml/min/mmHg)

**FIGURE 10:**
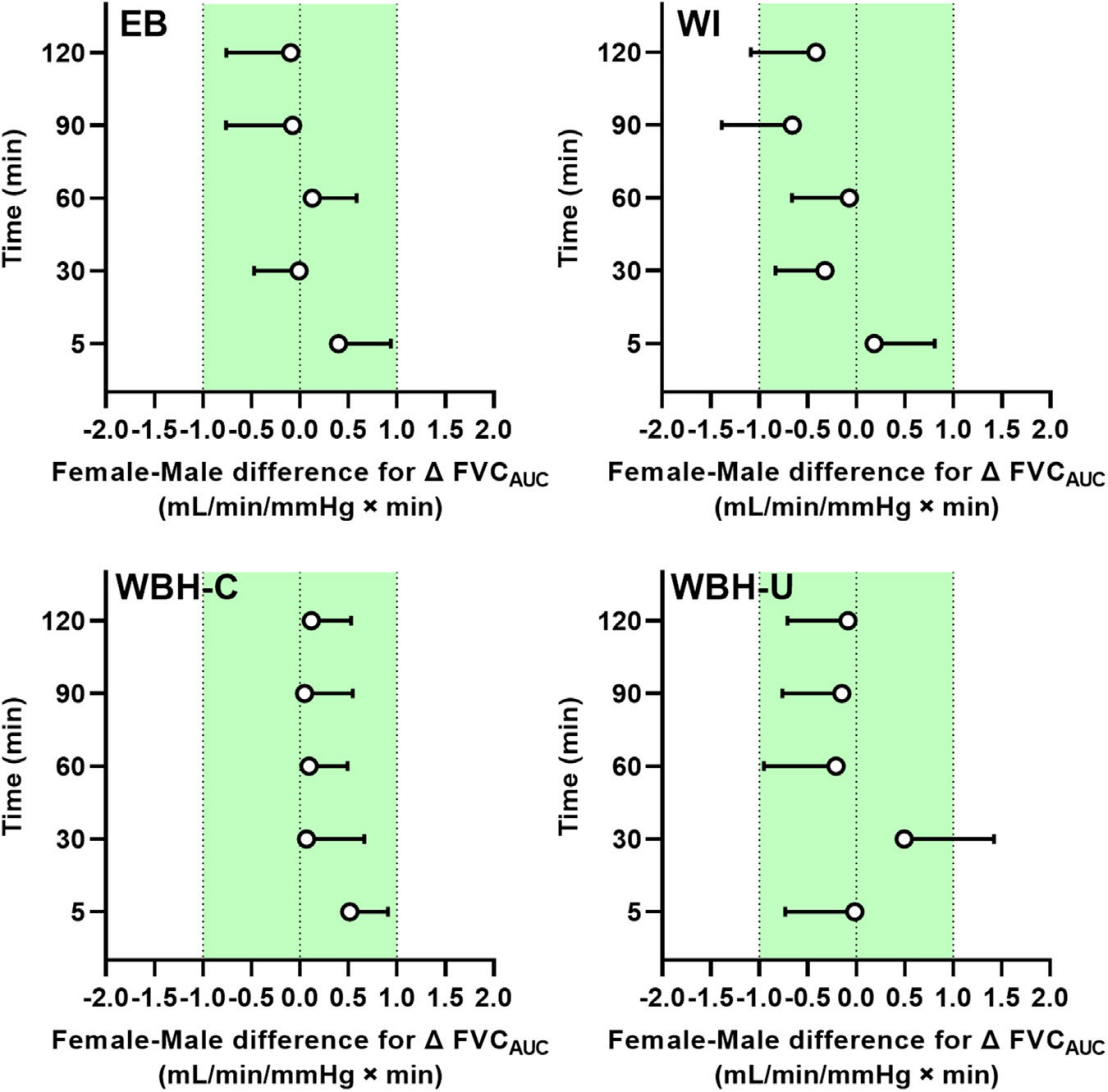
Female–male difference for change in forearm vascular conductance area under the curve (FVC_AUC_) during reactive hyperaemia in response to limb and whole‐body heating modalities. Data are presented as the mean difference (females minus males) with upper 90% confidence interval after forearm heating with an electric blanket (EB, 11 males and seven females), forearm hot water immersion (WI, 11 males and nine females), whole‐body heating with the arm covered (WBH‐C, 11 males and eight females) and whole‐body heating with the arm uncovered (WBH‐U, 11 males and seven females). The green shading depicts the equivalence limit (1 ml/min/mmHg × min)

When considering the female–male difference for change in FMD (Figure 8), the upper 90% confidence interval fell outside the equivalence limit (3%) during electric blanket heating (at 30 and 60 min), forearm water immersion (at all time points), whole‐body heating with the arm covered (at all time points) and whole‐body heating with the arm uncovered (at ≤5, 30, 90 and 120 min when calculated from pre‐occlusion diameter and at ≤5, 60, 90, and 120 min when calculated from end‐occlusion diameter). When considering the female–male difference for change in peak forearm vascular conductance during PORH (Figure 9), the upper 90% confidence interval fell outside the equivalence limit (1 ml/min/mmHg) during electric blanket heating (at ≤5 min), forearm water immersion (at ≤5 and 90 min), whole‐body heating with the arm covered (at ≤5, 30, 60 and 90 min) and whole‐body heating with the arm uncovered (at ≤5, 60, 90 and 120 min). When considering the female–male difference for change in forearm vascular conductance area under the curve during PORH (Figure 10), the upper 90% confidence interval fell outside the equivalence limit (1 ml/min/mmHg × min) during forearm water immersion (at 90 and 120 min) and whole‐body heating with the arm uncovered (at 30 min).

## 4 DISCUSSION

The overall objective of this study was to gain a better understanding of the acute effect of heat exposure on brachial artery FMD and PORH. The main findings are as follows: (1) any acute change in brachial artery FMD or PORH following heating is transient and short‐lasting (≤60 min); (2) whole‐body heating leads to an immediate decrease in brachial artery FMD, whereas forearm heating leads to an immediate increase in FMD; (3) the acute effect of whole‐body heating on brachial artery FMD and PORH does not differ if the forearm is exposed or not exposed to the heating stimulus; and (4) the acute change in brachial artery FMD and PORH following limb and whole‐body heating does not differ statistically between males and females, although the observed differences were not always equivalent.

The first objective of the present study was to characterize the time course of any acute change in brachial artery FMD and PORH following heat exposure. The results demonstrate that any change (increase or decrease) in FMD and PORH following heat exposure is transient and resolves within 60 min. One exception was an increase in FMD following forearm hot water immersion that persisted until 90 min post‐heating. Nonetheless, the greatest changes in brachial artery FMD and PORH were generally observed within the first 30 min post‐heating. The acute change in FMD does not appear to be driven by changes in baseline diameter and/or the shear stimulus for dilatation (SR_AUC_), because a significant effect of time was also observed when FMD values were adjusted for these variables. This time course characterization builds upon previous studies that measured FMD at a single time point after heat exposure and is consistent with the informal observation that acute changes in FMD have generally been observed when measurements are performed within ∼30–45 min post‐heating (Cheng et al., 2019, 2021; Coombs et al., 2021; Romero et al., 2017; Tinken et al., 2009), whereas no change is observed when measurements are performed ∼45–60 min post‐heating (Alali et al., 2020; Behzadi et al., 2022; Brunt, Jeckell et al., 2016; Engelland et al., 2020; Gravel et al., 2019). Although it was not the specific objective of this study, the results provide a basis for future studies to perform brachial artery FMD measurements within ∼30–45 min post‐heating to study how various factors might modulate the acute effect of heat exposure on macrovascular function. That said, the time at which an increase in FMD occurred was inconsistent between heating modalities. In response to limb heating, an increase in FMD was observed 30 min following electric blanket heating, whereas an increase was observed 30 and 90 min following forearm hot water immersion. In response to whole‐body heating, an immediate decrease in FMD was observed when it was calculated using pre‐occlusion diameter, whereas an increase was observed ≤5 and 30 min following both whole‐body heating modalities when calculated using end‐occlusion diameter. Current expert guidelines recommend calculating FMD from pre‐occlusion dimeter (Thijssen et al., 2019). Although a previous study calculated FMD from end‐occlusion diameter to account for the large dilatation that occurs during whole‐body heating (Coombs et al., 2021), the physiological significance (if any) of this approach remains unknown. Regardless, the acute effect of whole‐body heating on brachial artery FMD was short lasting and resolved within 30 min. However, we cannot rule out the possibility that a protracted increase in brachial artery FMD might be observed beyond 2 h post‐heating. Indeed, some evidence suggests that popliteal artery FMD is increased 24 h after 60 min of hot water immersion (Didier et al., 2022).

For PORH, we observed an increase in peak forearm vascular conductance during reactive hyperaemia immediately (≤5 min) after forearm hot water immersion and both whole‐body heating modalities. Although this increase might reflect improved microvascular function, the greater peak values are probably attributable to greater pre‐occlusion forearm vascular conductance values. Indeed, there was no change in forearm vascular conductance area under the curve in response to each heating modality. Most (Brunt, Jeckell et al., 2016; Coombs et al., 2019; Engelland et al., 2020; Romero et al., 2017), but not all (Cheng et al., 2021) previous studies performed on young healthy adults in which microvascular function was quantified as vascular conductance did not observe an acute effect of heating on microvascular function. The present results extend these previous observations and suggest that limb and whole‐body heating do not appear to modulate forearm microvascular function acutely in young healthy adults.

The second objective of the present study was to determine whether limb and whole‐body heating lead to a different acute change in brachial artery FMD and/or PORH. The results show that, when calculated using pre‐occlusion diameter, whole‐body heating results in an immediate decrease in brachial artery FMD, whereas limb heating tends to increase FMD. The immediate decrease in brachial artery FMD after whole‐body heating is likely related to the large increase in diameter that occurs during heating. As a consequence, a considerable decrease in brachial artery diameter was observed during the occlusion period. In addition, the shear stimulus for dilatation (SR_AUC_) was reduced immediately post‐heating, and whole‐body heating is likely to elicit greater sympathoexcitation relative to limb heating that, on its own, reduces brachial artery FMD (Atkinson et al., 2015). Similar responses were observed by Alali et al. (2020) within the radial artery when an FMD measurement was performed during whole‐body heating. When calculated using end‐occlusion diameter, whole‐body heating increased brachial artery FMD ≤5 and 30 min following whole‐body heating. This observation is consistent with the study by Coombs et al. (2021) that reported an increase in FMD ∼10 min following whole‐body heating when it was calculated from end‐occlusion dimeter (FMD calculated from pre‐occlusion diameter was not reported). However, the physiological relevance of calculating FMD from end‐occlusion diameter remains unknown. For this reason, we conclude that the immediate (≤30 min) effect of heat exposure on brachial artery FMD differs between limb and whole‐body heating modalities. That said, a direct comparison between the limb and whole‐body heating modalities used is limited by the fact that we did not match the duration of heating or the increase in core temperature. Furthermore, measurements were performed in the seated posture during forearm hot water immersion, in contrast to the supine posture during whole‐body heating. Although this resulted in different absolute preheating FMD values, these differences were accounted for by analysing the subsequent change in FMD from preheating values. For PORH, a greater peak forearm vascular conductance but lower area under the curve was observed immediately after whole‐body heating compared with limb heating. The greater peak forearm vascular conductance values are likely to reflect a greater pre‐occlusion forearm vascular conductance, whereas the lower area under the curve is likely to be related to the aforementioned decrease in diameter that occurs during occlusion following whole‐body heating. Beyond these acute effects, there were no differences in peak or area under the curve for forearm vascular conductance between limb and whole‐body heating modalities. Accordingly, limb and whole‐body heating do not appear to have a different acute effect upon forearm microvascular function during PORH.

The third objective of the present study was to determine whether direct limb heating is required to observe an acute increase in brachial artery FMD and PORH following whole‐body heating. We hypothesized that direct forearm heating would lead to a greater change in brachial artery FMD and PORH following whole‐body heating, based on the observation that an increase in skin temperature is required for cutaneous microvascular adaptations following repeated lower‐limb heating (Carter, Spence, Atkinson, Pugh, Naylor et al., 2014). In contrast to our hypothesis, the acute effect of whole‐body heating on brachial artery FMD and PORH did not differ when the forearm was exposed or unexposed to the heating stimulus. Although exposure of the forearm to the heating stimulus resulted in a greater increase in forearm skin temperature, the magnitude of this increase (∼3°C) might have been insufficient to mediate a different change in brachial artery FMD or PORH. Indeed, exposing the forearm to the heating stimulus did not result in a greater shear rate during whole‐body heating. Further evidence against the premise that direct limb heating is required to observe an acute improvement in FMD and PORH following heat exposure has recently been provided by Cheng et al. (2021), who observed a greater brachial artery FMD and peak forearm vascular conductance during PORH immediately after 45 min of lower‐leg hot water immersion.

The fourth objective of the present study was to determine whether biological sex modulates the acute effect of heat exposure on brachial artery FMD and PORH. To our knowledge, this is the first attempt to evaluate potential sex‐based differences in the acute effect of heat exposure on brachial artery macro‐ and microvascular function. We hypothesized that greater changes in FMD and PORH would be observed in females, owing to potentially greater increases in core temperature and shear rate during heat exposure (Larson et al., 2021). In the present study, the change in core, mean skin and forearm skin temperatures did not differ between males and females during each heating modality, whereas a greater increase in shear rate was observed in females during forearm hot water immersion and both whole‐body heating modalities. Nonetheless, the change in brachial artery FMD and PORH did not differ statistically between males and females. However, these results might be limited by a lack of statistical power, because we did not power the study to detect potential sex‐based differences. For this reason, we also considered whether the changes in brachial artery FMD and PORH could be considered equivalent between males and females. Despite the lack of statistical differences, the upper 90% confidence interval for the female–male difference for change in FMD fell outside the equivalence limit at several time points during each heating modality. In general, a greater change (decrease or increase) in brachial artery FMD was observed in females compared with males, although a consistent trend was not apparent from the results (Figure 8). Several female–male differences for change in peak forearm vascular conductance also fell outside the upper 90% confidence interval during each heating modality. As for FMD, a greater change in females was generally observed although no clear pattern emerges from the results (Figure 9). In contrast, most female–male differences fell within the upper 90% confidence interval for forearm vascular conductance area under the curve (Figure 10). Taken together, our interpretation of these findings is that females might display a greater acute change in brachial artery FMD and/or peak forearm vascular conductance during PORH following heat exposure, and the results provide a basis for future studies to investigate this possibility.

### 4.1 Limitations

Some limitations must be considered when interpreting the present results. First, the sample size was determined from expected pre–post changes in brachial artery FMD. Accordingly, the study was not specifically powered to test interactions, and larger, more focused studies are warranted to confirm or refute the observed differences between limb and whole‐body heating modalities and between males and females. Second, the findings are specific to the brachial artery, because we did not perform measurements at other commonly used sites, such as the femoral and/or popliteal arteries. Third, we recruited young, healthy adults and cannot rule out the possibility that the acute effect of heat exposure might differ in other populations. Fourth, blood samples were not taken, which prevents us from calculating shear stress. Fifth, we did not perform a measurement of endothelium‐independent vasodilatation; therefore, we cannot rule out a potential contribution of alterations in vascular smooth muscle sensitivity to the observed changes in FMD. Lastly, female participants were studied during the follicular phase of the menstrual cycle or in the absence of a regular cycle. We therefore cannot determine whether menstrual cycle phase might modulate the acute effect of heat exposure on brachial artery FMD and/or PORH.

### 4.2 Perspectives

There is considerable interest in the potential cardiovascular health benefits of heat exposure. This interest is motivated, in part, by the effect of repeated heat exposure on macro‐ and microvascular function, measured by the FMD and PORH techniques, respectively. To study how heat exposure modulates vascular function, several studies have considered acute changes following various heating modalities. Such studies provide insights into the factors that might mediate longer‐term adaptations in response to repeated heat exposure (Romero et al., 2022), and they are expected to continue because there are several unanswered questions regarding the effect of heat exposure on vascular function, such as (Brunt & Minson, 2021): the underlying physiological modulators (e.g., shear stress vs. heat shock proteins vs. circulating factors), population differences (e.g., males/females, age, disease) and/or optimal heating parameters (e.g., duration, intensity). To date, the acute effect of heat exposure on brachial artery macro‐ and microvascular function has proved variable. The present study provides four novel observations that can help to explain this variability. First, the study demonstrates that the timing of post‐heating measurements is important and that any change in vascular function is likely to be transient and short lasting. Second, whole‐body heating leads to an immediate decrease in brachial artery FMD, whereas limb heating leads to an increase in FMD. Accordingly, use of a limb heating modality seems more appropriate if the objective is to study how heat exposure improves macrovascular function acutely. Third, direct forearm heating does not alter the acute effect of whole‐body heat exposure on brachial artery FMD and PORH. Fourth, consideration should be given to reporting sex‐disaggregated data and/or to ensuring an adequate sample size to test for sex‐based differences.

## 5 CONCLUSION

This study demonstrates that, in young healthy adults: (1) any acute change in brachial artery FMD and PORH after heat exposure is transient and short lasting (≤60 min); (2) whole‐body heating leads to an immediate decrease in brachial artery FMD, whereas limb heating leads to a transient increase in FMD; (3) forearm and whole‐body heating generally do not exert an acute effect on forearm vascular conductance during PORH; (4) the acute effect of whole‐body heating on brachial artery FMD and PORH does not differ when the forearm is exposed or unexposed to the heating stimulus; and (5) the acute change in brachial artery FMD and PORH following limb and whole‐body heating does not differ statistically between males and females, but the differences are not always equivalent. These findings further our understanding of the acute effect of heat exposure on brachial artery macro‐ and microvascular function and provide a useful basis for future studies investigating the acute effect of heat exposure on vascular function.

## AUTHOR CONTRIBUTIONS

The experiments were performed within the Human Integrative Physiology Laboratory at Centre ÉPIC of the Montreal heart Institute. Georgia K. Chaseling, Hugo Gravel, Nicholas Ravanelli and Daniel Gagnon contributed to the conception of the work. All authors contributed to the acquisition, analysis or interpretation of data for the work and to the drafting of the work or revising it critically for important intellectual. All authors approved the final version of the manuscript and agree to be accountable for all aspects of the work in ensuring that questions related to the accuracy or integrity of any part of the work are appropriately investigated and resolved. All persons designated as authors qualify for authorship, and all those who qualify for authorship are listed.

## CONFLICT OF INTEREST

None declared.

## Supporting information

Statistical Summary Document

## Data Availability

The original data from this work are available upon request to the corresponding author.

## References

[eph13287-bib-0001] Akerman, A. P. , Thomas, K. N. , van Rij, A. M. , Body, E. D. , Alfadhel, M. , & Cotter, J. D. (2019). Heat therapy vs. supervised exercise therapy for peripheral arterial disease: A 12‐wk randomized, controlled trial. American Journal of Physiology. Heart and Circulatory Physiology, 316(6), H1495–H1506. 31002283 10.1152/ajpheart.00151.2019

[eph13287-bib-0002] Alali, M. H. , Vianna, L. C. , Lucas, R. A. I. , Junejo, R. T. , & Fisher, J. P. (2020). Impact of whole body passive heat stress and arterial shear rate modification on radial artery function in young men. Journal of Applied Physiology, 129(6), 1373–1382. 33031019 10.1152/japplphysiol.00296.2020

[eph13287-bib-0003] Atkinson, C. L. , Lewis, N. C. , Carter, H. H. , Thijssen, D. H. , Ainslie, P. N. , & Green, D. J. (2015). Impact of sympathetic nervous system activity on post‐exercise flow‐mediated dilatation in humans. The Journal of Physiology, 593(23), 5145–5156. 26437709 10.1113/JP270946 PMC4666994

[eph13287-bib-0004] Atkinson, G. (2014). Shear rate normalization is not essential for removing the dependency of flow‐mediated dilation on baseline artery diameter: past research revisited. Physiological Measurement, 35(9), 1825–1835. 25139144 10.1088/0967-3334/35/9/1825

[eph13287-bib-0005] Bailey, T. G. , Cable, N. T. , Miller, G. D. , Sprung, V. S. , Low, D. A. , & Jones, H. (2016). Repeated warm water immersion induces similar cerebrovascular adaptations to 8 weeks of moderate‐intensity exercise training in females. International Journal of Sports Medicine, 37, 757–765. 27286178 10.1055/s-0042-106899

[eph13287-bib-0006] Behzadi, P. , Ravanelli, N. , Gravel, H. , Barry, H. , Debray, A. , Chaseling, G. K. , Jacquemet, V. , Neagoe, P. E. , Nigam, A. , Carpentier, A. C. , Sirois, M. G. , & Gagnon, D. (2022). Acute effect of passive heat exposure on markers of cardiometabolic function in adults with type 2 diabetes mellitus. Journal of Applied Physiology, 132(5), 1154–1166. 35323077 10.1152/japplphysiol.00800.2021

[eph13287-bib-0007] Brunt, V. E. , Howard, M. J. , Francisco, M. A. , Ely, B. R. , & Minson, C. T. (2016). Passive heat therapy improves endothelial function, arterial stiffness and blood pressure in sedentary humans. The Journal of Physiology, 594(18), 5329–5342. 27270841 10.1113/JP272453 PMC5023696

[eph13287-bib-0008] Brunt, V. E. , Jeckell, A. T. , Ely, B. R. , Howard, M. J. , Thijssen, D. H. , & Minson, C. T. (2016). Acute hot water immersion is protective against impaired vascular function following forearm ischemia‐reperfusion in young healthy humans. American Journal of Physiology. Regulatory, Integrative and Comparative Physiology, 311(6), R1060–R1067. 27707723 10.1152/ajpregu.00301.2016 PMC6195651

[eph13287-bib-0009] Brunt, V. E. , & Minson, C. T. (2021). Heat therapy: mechanistic underpinnings and applications to cardiovascular health. Journal of Applied Physiology, 130(6), 1684–1704. 33792402 10.1152/japplphysiol.00141.2020 PMC8285605

[eph13287-bib-0010] Carter, H. H. , Spence, A. L. , Atkinson, C. L. , Pugh, C. J. , Cable, N. T. , Thijssen, D. H. , Naylor, L. H. , & Green, D. J. (2014). Distinct effects of blood flow and temperature on cutaneous microvascular adaptation. Medicine and Science in Sports and Exercise, 46(11), 2113–2121. 25338190 10.1249/MSS.0000000000000349

[eph13287-bib-0011] Carter, H. H. , Spence, A. L. , Atkinson, C. L. , Pugh, C. J. , Naylor, L. H. , & Green, D. J. (2014). Repeated core temperature elevation induces conduit artery adaptation in humans. European Journal of Applied Physiology, 114(4), 859–865. 24399113 10.1007/s00421-013-2817-2

[eph13287-bib-0012] Cheng, J. L. , Au, J. S. , & MacDonald, M. J. (2019). Peripheral artery endothelial function responses to altered shear stress patterns in humans. Experimental Physiology, 104(7), 1126–1135. 30993773 10.1113/EP087597

[eph13287-bib-0013] Cheng, J. L. , & MacDonald, M. J. (2019). Effect of heat stress on vascular outcomes in humans. Journal of Applied Physiology, 126(3), 771–781. 30676869 10.1152/japplphysiol.00682.2018 PMC6459390

[eph13287-bib-0014] Cheng, J. L. , Williams, J. S. , Hoekstra, S. P. , & MacDonald, M. J. (2021). Improvements in vascular function in response to acute lower limb heating in young healthy males and females. Journal of Applied Physiology, 131(1), 277–289. 34013754 10.1152/japplphysiol.00630.2020

[eph13287-bib-0015] Coombs, G. B. , Barak, O. F. , Phillips, A. , Mijacika, T. , Sarafis, Z. , HXL, A. , Squair, J. W. , Bammert, T. D. , DeSouza, N. M. , Gagnon, D. , Krassioukov, A. V. , Dujic, Z. , DeSouza, C. A. , & Ainslie, P. N. (2019). Acute heat stress reduces biomarkers of endothelial activation but not macro‐ or microvascular dysfunction in cervical spinal cord injury. American Journal of Physiology. Heart and Circulatory Physiology, 316(3), H722–H733. 30575438 10.1152/ajpheart.00693.2018 PMC6459313

[eph13287-bib-0016] Coombs, G. B. , Tremblay, J. C. , Shkredova, D. A. , Carr, J. , Wakeham, D. J. , Patrician, A. , & Ainslie, P. N. (2021). Distinct contributions of skin and core temperatures to flow‐mediated dilation of the brachial artery following passive heating. Journal of Applied Physiology, 130(1), 149–159. 33119469 10.1152/japplphysiol.00502.2020

[eph13287-bib-0017] Didier, K. D. , Hammer, S. M. , Alexander, A. M. , Rollins, K. S. , & Barstow, T. J. (2022). The acute effects of passive heating on endothelial function, muscle microvascular oxygen delivery, and expression of serum HSP90α. Microvascular Research, 142, 104356. 35276210 10.1016/j.mvr.2022.104356

[eph13287-bib-0018] Engelland, R. E. , Hemingway, H. W. , Tomasco, O. G. , Olivencia‐Yurvati, A. H. , & Romero, S. A. (2020). Acute lower leg hot water immersion protects macrovascular dilator function following ischaemia‐reperfusion injury in humans. Experimental Physiology, 105(2), 302–311. 31707732 10.1113/EP088154 PMC7429992

[eph13287-bib-0019] Gravel, H. , Behzadi, P. , Cardinal, S. , Barry, H. , Neagoe, P. E. , Juneau, M. , Nigam, A. , Sirois, M. G. , & Gagnon, D. (2020). Acute vascular benefits of Finnish sauna bathing in patients with stable coronary artery disease. Canadian Journal of Cardiology, 37(3), 493–499. 32615263 10.1016/j.cjca.2020.06.017

[eph13287-bib-0020] Gravel, H. , Coombs, G. B. , Behzadi, P. , Marcoux‐Clément, V. , Barry, H. , Juneau, M. , Nigam, A. , & Gagnon, D. (2019). Acute effect of Finnish sauna bathing on brachial artery flow‐mediated dilation and reactive hyperemia in healthy middle‐aged and older adults. Physiological Reports, 7(13), e14166. 31293098 10.14814/phy2.14166 PMC6640592

[eph13287-bib-0021] Imamura, M. , Biro, S. , Kihara, T. , Yoshifuku, S. , Takasaki, K. , Otsuji, Y. , Minagoe, S. , Toyama, Y. , & Tei, C. (2001). Repeated thermal therapy improves impaired vascular endothelial function in patients with coronary risk factors. Journal of the American College of Cardiology, 38(4), 1083–1088. 11583886 10.1016/s0735-1097(01)01467-x

[eph13287-bib-0022] Kihara, T. , Biro, S. , Imamura, M. , Yoshifuku, S. , Takasaki, K. , Ikeda, Y. , Otuji, Y. , Minagoe, S. , Toyama, Y. , & Tei, C. (2002). Repeated sauna treatment improves vascular endothelial and cardiac function in patients with chronic heart failure. Journal of the American College of Cardiology, 39(5), 754–759. 11869837 10.1016/s0735-1097(01)01824-1

[eph13287-bib-0023] Larson, E. A. , Ely, B. R. , Brunt, V. E. , Francisco, M. A. , Harris, S. M. , Halliwill, J. R. , & Minson, C. T. (2021). Brachial and carotid hemodynamic response to hot water immersion in men and women. American Journal of Physiology. Regulatory, Integrative and Comparative Physiology, 321(6), R823–r832. 34643115 10.1152/ajpregu.00110.2021 PMC8714810

[eph13287-bib-0024] Lee, J. Y. , Wakabayashi, H. , Wijayanto, T. , & Tochihara, Y. (2010). Differences in rectal temperatures measured at depths of 4–19 cm from the anal sphincter during exercise and rest. European Journal of Applied Physiology, 109(1), 73–80. 19787368 10.1007/s00421-009-1217-0

[eph13287-bib-0025] McGarity‐Shipley, E. C. , Schmitter, S. M. , Williams, J. S. , King, T. J. , McPhee, I. A. C. , & Pyke, K. E. (2021). The impact of repeated, local heating‐induced increases in blood flow on lower limb endothelial function in young, healthy females. European Journal of Applied Physiology, 121(11), 3017–3030. 34251539 10.1007/s00421-021-04749-7

[eph13287-bib-0026] Naylor, L. H. , Carter, H. , FitzSimons, M. G. , Cable, N. T. , Thijssen, D. H. , & Green, D. J. (2011). Repeated increases in blood flow, independent of exercise, enhance conduit artery vasodilator function in humans. American Journal of Physiology. Heart and Circulatory Physiology, 300(2), H664–H669. 21131471 10.1152/ajpheart.00985.2010

[eph13287-bib-0027] O'Brien, M. W. , & Kimmerly, D. S. (2022). Is “not different” enough to conclude similar cardiovascular responses across sexes? American Journal of Physiology. Heart and Circulatory Physiology, 322(3), H355–h358. 34995165 10.1152/ajpheart.00687.2021

[eph13287-bib-0028] Ramanathan, N. L. (1964). A new weighting system for mean surface temperature of the human body. Journal of Applied Physiology, 19(3), 531–533. 14173555 10.1152/jappl.1964.19.3.531

[eph13287-bib-0029] Romero, S. A. , Gagnon, D. , Adams, A. N. , Cramer, M. N. , Kouda, K. , & Crandall, C. G. (2017). Acute limb heating improves macro‐ and microvascular dilator function in the leg of aged humans. American Journal of Physiology. Heart and Circulatory Physiology, 312(1), H89–H97. 27836894 10.1152/ajpheart.00519.2016 PMC5283915

[eph13287-bib-0030] Romero, S. A. , Richey, R. E. , & Hemingway, H. W. (2022). Cardiovascular adjustments following acute heat exposure. Exercise and Sport Sciences Reviews, 50(4), 194–202. 36044739 10.1249/JES.0000000000000304 PMC9474635

[eph13287-bib-0031] Thijssen, D. H. J. , Bruno, R. M. , van Mil, A. , Holder, S. M. , Faita, F. , Greyling, A. , Zock, P. L. , Taddei, S. , Deanfield, J. E. , Luscher, T. , Green, D. J. , & Ghiadoni, L. (2019). Expert consensus and evidence‐based recommendations for the assessment of flow‐mediated dilation in humans. European Heart Journal, 40(30), 2534–2547. 31211361 10.1093/eurheartj/ehz350

[eph13287-bib-0032] Thomas, K. N. , van Rij, A. M. , Lucas, S. J. , Gray, A. R. , & Cotter, J. D. (2016). Substantive hemodynamic and thermal strain upon completing lower‐limb hot‐water immersion; comparisons with treadmill running. Temperature, 3(2), 286–297. 10.1080/23328940.2016.1156215 PMC4964998 27857958

[eph13287-bib-0033] Tinken, T. M. , Thijssen, D. H. , Hopkins, N. , Black, M. A. , Dawson, E. A. , Minson, C. T. , Newcomer, S. C. , Laughlin, M. H. , Cable, N. T. , & Green, D. J. (2009). Impact of shear rate modulation on vascular function in humans. Hypertension, 54(2), 278–285. 19546374 10.1161/HYPERTENSIONAHA.109.134361 PMC3012006

