# Supplementary material for: The acute effect of heat exposure on forearm macro‐ and microvascular function: Impact of measurement timing, heating modality and biological sex
Source: Exp Physiol. 2022 Dec 19;108(2):221–39. doi: 10.1113/EP090732 (PMC10103856; doi:10.1113/EP090732)
Supplement: Supplementary file 1 — Statistical Summary Document [file EPH-108-221-s001.docx]

**Manuscript Title:** The acute effect of heat exposure on forearm macro- and microvascular function: Impact of measurement timing, heating modality, and biological sex.

**Authors:** Georgia K. Chaseling, Amélie Debray, Hugo Gravel, Nicholas Ravanelli, Audrey-Ann Bartlett, Daniel Gagnon

**Animal model used, if applicable:** n/a

**Underlying hypothesis:** We tested the hypothesis that: 1) heat exposure leads to a rapid and transient improvement in brachial artery FMD; 2) the magnitude of change in brachial artery FMD is greater following limb vs. whole-body heating; 3) a greater change in brachial artery FMD would be observed when the forearm is directly exposed to the heating stimulus during whole-body heating; 4) females display a greater change in brachial artery FMD following heat exposure.

**Definitions of ‘n’:** number of participants

**Statistical summary table:**

| Experimental question number* | Finding/ conclusion | Experimental location/ variable | Mean value | SD | n val. | P** | Units | Data comparisons | Statistical test | Any other variable  e.g. subjects’ age or sex | Figure/ table in which data are presented |
| --- | --- | --- | --- | --- | --- | --- | --- | --- | --- | --- | --- |
| 1. Temporal change in FMD | Limb and whole-body heating exert a transient change in brachial artery FMD | EB | 5 min: 0.64  30 min: 2.15  60 min: 1.36  90 min: 0.66  120 min: 1.15 | 1.83  2.74  2.65  1.98  1.99 | 17  17  16  16  17 | 0.4902  **0.0209**  0.1888  0.5459  0.1101 | %FMD (change from pre-heating) | 5, 30, 60, 90, 120 min vs. pre-heating | Mixed effects model with Dunnett multiple comparisons | 11 males: 30 ± 6 years, 70 ± 9 kg, 1.7 ± 0.1 m, 22.3 ± 2.3 kg/m^2^  9 females: 25 ± 4 years, 57 ± 7 kg, 1.6 ± 0.0 m, 20.1 ± 3.1 kg/m^2^ | Table 1 and figure 5 |
|  |  | WI | 5 min: 2.29  30 min: 3.14  60 min: 2.38  90 min: 2.89  120 min: 1.23 | 5.54  3.41  4.31  4.28  3.22 | 18  20  18  18  19 | 0.2924  **0.0026**  0.1044  **0.0368**  0.3474 |  |  |  |  |  |
|  |  | WBH-C | 5 min: -8.29  30 min: -1.65  60 min: 0.12  90 min: -0.13  120 min: -0.32 | 4.17  5.84  3.50  5.00  4.04 | 19  19  19  19  18 | **<0.0001**  0.6214  0.9998  0.9999  0.9963 |  |  |  |  |  |
|  |  | WBH-U | 5 min: -6.04  30 min: 0.53  60 min: 1.91  90 min: 0.49  120 min: 0.51 | 4.02  3.88  3.06  2.48  2.03 | 18  18  18  17  17 | **<0.0001**  0.9663  0.0704  0.8746  0.7301 |  |  |  |  |  |
| 2. Effect of heating modality on acute change in FMD | Limb heating transiently increases FMD whereas whole-body heating transiently decreases FMD. | 5 min | EB: 0.64  WI: 2.29  WBH-C: -8.29  WBH-U : -6.04 | 1.83  5.54  4.17  4.02 | 17  18  19  18 | WI vs. EB: 0.4075  **WBH-C vs. EB: <0.0001**  **WBH-U vs. EB:** **<0.0001**  **WBH-C vs. WI: <0.0001**  **WBH-U vs. WI: 0.0001**  WBH-U vs. WBH-C: 0.3533 | %FMD (change from pre-heating) | EB vs. WI vs. WBH-C vs. WBH-U | Mixed effects model with Tukey multiple comparisons | 11 males: 30 ± 6 years, 70 ± 9 kg, 1.7 ± 0.1 m, 22.3 ± 2.3 kg/m^2^  9 females: 25 ± 4 years, 57 ± 7 kg, 1.6 ± 0.0 m, 20.1 ± 3.1 kg/m^2^ | Figure 5 |
|  |  | 30 min | EB: 2.15  WI: 3.14  WBH-C: -1.65  WBH-U: 0.53 | 2.74  3.41  5.84  3.88 | 17  20  19  18 | WI vs. EB: 0.7775  **WBH-C vs. EB: 0.0167**  WBH-U vs. EB: 0.1111  **WBH-C vs. WI: 0.0349**  WBH-U vs. WI: 0.2713  WBH-U vs. WBH-C: 0.3171 |  |  |  |  |  |
|  |  | 60 min | EB: 1.36  WI: 2.38  WBH-C: 0.12  WBH-U: 1.91 | 2.65  4.31  3.50  3.06 | 16  20  19  18 | WI vs. EB: 0.7115  WBH-C vs. EB: 0.3830  WBH-U vs. EB: 0.8863  WBH-C vs. WI: 0.4421  WBH-U vs. WI: 0.9848  WBH-U vs. WBH-C: 0.0938 |  |  |  |  |  |
|  |  | 90 min | EB: 0.66  WI: 2.89  WBH-C: -0.13  WBH-U: 0.49 | 1.98  4.28  5.00  2.48 | 16  18  19  17 | WI vs. EB: 0.2575  WBH-C vs. EB: 0.8996  WBH-U vs. EB: 0.9965  WBH-C vs. WI: 0.2237  WBH-U vs. WI: 0.2092  WBH-U vs. WBH-C: 0.9549 |  |  |  |  |  |
|  |  | 120 min | EB: 1.15  WI: 1.23  WBH-C: -0.32  WBH-U: 0.51 | 1.99  3.22  4.04  2.03 | 17  19  18  17 | WI vs. EB: 0.9994  WBH-C vs. EB: 0.4963  WBH-U vs. EB: 0.7193  WBH-C vs. WI: 0.5704  WBH-U vs. WI: 0.8588  WBH-U vs. WBH-C: 0.8045 |  |  |  |  |  |
| 3. Effect of forearm heating during whole-body heating on acute change in FMD | The acute effect of whole-body heating does not differ if the arm is exposed or unexposed to the heating stimulus. | 5 min | WBH-C: -8.29  WBH-U : -6.04 | 4.17  4.02 | 19  18 | 0.3533 | %FMD (change from pre-heating) | WBH-C vs. WBH-U | Mixed effects model with Tukey multiple comparisons | 11 males: 30 ± 6 years, 70 ± 9 kg, 1.7 ± 0.1 m, 22.3 ± 2.3 kg/m^2^  9 females: 25 ± 4 years, 57 ± 7 kg, 1.6 ± 0.0 m, 20.1 ± 3.1 kg/m^2^ | Figure 5 |
|  |  | 30 min | WBH-C: -1.65  WBH-U: 0.53 | 5.84  3.88 | 19  18 | 0.3171 |  |  |  |  |  |
|  |  | 60 min | WBH-C: 0.12  WBH-U: 1.91 | 3.50  3.06 | 19  18 | 0.0938 |  |  |  |  |  |
|  |  | 90 min | WBH-C: -0.13  WBH-U: 0.49 | 5.00  2.48 | 19  17 | 0.9549 |  |  |  |  |  |
|  |  | 120 min | WBH-C: -0.32  WBH-U: 0.51 | 4.04  2.03 | 18  17 | 0.8045 |  |  |  |  |  |
| 4. Does the acute effect of heat exposure on FMD differ between males and females? | Acute changes in FMD do not differ between males (M) and females (F) | EB | 5 min  M: 0.57  F: 0.74  30 min  M: 1.48  F: 3.12  60 min  M: 0.63  F: 2.29  90 min  M: 1.02  F: 0.06  120 min  M:0.94  F: 1.44 | 0.57  0.74  1.48  3.12  0.63  2.29  1.02  0.06  0.94  1.44 | 10  7  10  7  9  7  10  6  10  7 | 0.8796  0.1435  0.1238  0.4480  0.6485 | %FMD (change from pre-heating) | Males vs. females | Mixed effects model with Fisher’s LSD multiple comparisons | 11 males: 30 ± 6 years, 70 ± 9 kg, 1.7 ± 0.1 m, 22.3 ± 2.3 kg/m^2^  9 females:  25 ± 4 years, 57 ± 7 kg, 1.6 ± 0.0 m, 20.1 ± 3.1 kg/m^2^ | Figures 5 and 8 |
|  |  | WI | 5 min  M: 1.61  F: 3.37  30 min  M: 4.01  F: 2.08  60 min  M: 0.98  F: 4.58  90 min  M: 3.684  F: 2.086  120 min  M: 1.49  F: 0.88 | 4.83  6.78  3.62  2.99  4.30  3.57  4.26  4.39  2.24  4.39 | 11  7  11  9  11  7  9  9  11  8 | 0.2717  0.3089  0.2251  0.3792  0.7223 |  |  |  |  |  |
|  |  | WBH-C | 5 min  M: -7.45  F: -9.45  30 min  M: -1.39  F: -2.01  60 min  M: 0.27  F: -0.09  90 min  M: -1.08  F: 1.18  120 min  M: -1.16  F: 0.99 | 3.35  5.11  4.12  7.95  2.24  4.92  3.23  6.78  2.74  5.52 | 11  8  11  8  11  8  11  8  11  7 | 0.3593  0.7747  0.8677  0.3002  0.1920 |  |  |  |  |  |
|  |  | WBH-U | 5 min  M: -4.42  F: -8.60  30 min  M: -0.07  F: 1.46  60 min  M: 1.73  F: 2.25  90 min  M: 0.12  F: 1.17  120 min  M: 0.02  F: 1.29 | 3.47  3.64  4.12  3.56  1.52  5.00  2.22  2.98  2.35  1.15 | 11  7  11  7  11  6  11  6  11  7 | **0.0066**  0.3127  0.8209  0.4141  0.4001 |  |  |  |  |  |

*You may use multiple lines for the same question to indicate multiple comparisons

** Authors may wish to make the text bold where p is considered significant against a stated confidence limit.
